# Developing a psychological test battery to measure cognition in daily life

**DOI:** 10.3758/s13428-026-03128-4

**Published:** 2026-07-24

**Authors:** Andrew J. Aschenbrenner, Muchen Xi, Jeremy Cohn, Derek A. Simon, Joshua J. Jackson

**Affiliations:** 1 University of Kansas Alzheimer’s Disease Research Center, University of Kansas Medical Center, 4350 Shawnee Mission Parkway, Fairway, KS 66205 USA; 2 Department of Neurology, University of Kansas Medical Center, 4350 Shawnee Mission Parkway, Fairway, KS 66205 USA; 3 Department of Psychological and Brain Sciences, Washington University in St. Louis, 1 Brookings Drive, St. Louis, MO 63130 USA

**Keywords:** Remote, Cognition, High-frequency, Momentary, Contextual

## Abstract

Cognition is not a static process but is subject to substantial and meaningful variation within individuals over time. This has led to an increase in studies that aim to describe cognition in daily life by sampling participants repeatedly and remotely. Such studies, which we call high-frequency cognitive assessment or “HFCA”, tend to use a limited number of brief cognitive tests. This focus on a small number of tests leaves open questions concerning the psychometric characteristics of a wide array of cognitive tasks useful for HFCA that can guide researchers on appropriate task selection. We developed the Cognitive Variability Battery (CVB), a series of nine cognitive tests clustered into three distinct cognitive domains: attentional control, processing speed, and episodic memory. CVB was administered to participants three times per day for 3 weeks. Cognitive tests were rotated to reduce the length of any single testing session. Each test was administered up to 60 times. We provide detailed descriptions of the performance of each task, including variability, skew, and reliability statistics, using intraclass correlations. We examine the sensitivity of each test to several contextual factors including stress, affect, and social interactions. Finally, we provide power analyses on each cognitive test to determine how many assessments and participants are needed to detect effects of interest. These analyses will be essential to anyone seeking to implement HFCA testing in their own research programs, by providing guidance on which cognitive tests to select and how many participants and observations may be needed.

**Supplementary Information:**

The online version contains supplementary material available at https://doi.org/10.3758/s13428-026-03128-4.

## Introduction

It is widely accepted that cognitive processes are not static but can fluctuate substantially from assessment to assessment. As it has been well established now that these cognitive fluctuations are not noise, it is becoming common to capitalize on momentary cognitive variation as an additional indicator of the integrity of the cognitive system (Vaughan & Birney, 2023) by administering tests repeatedly over a short interval (e.g., multiple times across a week). Repeated assessment study designs go by many names (intensive longitudinal studies, ecological momentary assessment, ambulatory assessment, measurement bursts), all of which have slightly different connotations but reflect the same underlying principle of repeatedly sampling cognitive performance over a short period of time, typically remotely and in the participant’s lived environment, as opposed to in a laboratory or clinic setting.

In the present study, we use the atheoretical term “high-frequency cognitive assessment” (HFCA), but regardless of nomenclature, HFCA allows for the direct assessment of cognition as it occurs in daily life or in response to certain environmental stimuli. Performance on these repeated cognitive tests can then be examined in different ways. As a few examples, HFCA has been used to determine whether subtle effects of Alzheimer disease neuropathology are exaggerated at certain times of the day (Wilks et al., 2021), which individuals are most affected by environmental distractions (Madero et al., 2021) or social context (Cerino et al., 2021), and the influence of momentary loneliness (Kang et al., 2024), stress (Sliwinski et al., 2006), or motivation (Brose et al., 2012). There have even been demonstrations that daily cognition can correlate with meaningful functional outcomes such as risky driving behaviors (Aschenbrenner et al., 2025b).

In addition to examining the *level* of performance at any particular moment, cognitive scores from HFCA can also be quantified by examining the overall variability in scores across the testing period (e.g., using summary statistics such as the standard deviation). Multiple studies have now shown that high cognitive variability can be indicative of cognitive impairment (Aschenbrenner & Jackson, 2024); Cerino et al., 2021; Hultsch et al., 2000) or of genetic risk factors for neurodegenerative disorders such as Alzheimer disease (Aschenbrenner et al., 2024; Welhaf et al., 2023), even in the absence of overt cognitive deficits. Other studies have used cognitive variability to explore cognitive differences in healthy older versus younger adults and have shown that age effects depend heavily on the timescale of interest. For example, the standard deviation of reaction time across trials within a task increases in older adults (Anstey et al., 2005; Hultsch et al., 2002), yet older adults are typically less variable than younger adults across different days (Aschenbrenner & Jackson, 2024; Schmiedek et al., 2013), which may be attributed to many causes including differential learning and strategy use (Allaire & Marsiske, 2005). This latter interpretation is supported by the finding that performance variability is associated with practice-related gains in average performance over time (Allaire & Marsiske, 2005), that is, the most variable performers improved the most over the study. The idea is that some individuals identify when a specific response strategy is not effective (i.e., leads to low performance) and discard that strategy for a new (and possibly more effective) one, simultaneously increasing mean performance but also variability. The argument would then be that older adults are less able to “switch” strategies during testing and therefore have lower, but more consistent, performance across days (Hertzog et al., 2017; Shing et al., 2012).

Thus, it is clear that “single-shot” assessments of cognition obtained at a single time point, or even at widely spaced intervals, as in large-scale panel studies, are insufficient to fully characterize either an individual’s or a group’s cognitive ability, as they may have been measured on a day that does not describe their typical level of function (i.e., they may just be having a particularly good or bad day). Despite the numerous advantages of HFCA, such studies are hampered by one important limitation. Specifically, as participants must engage with the testing repeatedly (e.g., multiple times per day for 1 week or more), most researchers naturally opt to keep each individual testing session rather brief (i.e., 5 min or less) to minimize participant burden and maximize adherence. This can be especially important for “at-risk” populations such as individuals with severe depression or who have cognitive impairment, for whom engagement with repeated testing may be a rather stressful endeavor. These time constraints allow for, at most, a few measures to be given at each assessment. As a result, researchers are forced to choose between including several cognitive tests in their HFCA battery that each tap a different underlying cognitive domain (Cerino et al., 2021; Nicosia et al., 2022a), or including multiple indicators of only a single domain, such as episodic memory (Weizenbaum et al., 2025). While this limitation in test selection is an unfortunate necessity for the successful conduct of HFCA studies, it is potentially quite problematic, as it is not possible to evaluate whether observed cognitive fluctuations exist at the domain level or the task level. In other words, is poor performance on a test of memory on any particular occasion due to a momentary lapse in the efficiency of cognition broadly, or of memory processes per se*,* or due to something specific to the particular task that was selected (issues with stimuli selection or problematic instructions)?

Standard in-lab assessments are able to tease apart variability at different levels due to a long tradition of psychometric testing on each measure. As a result, it is well known how variables should covary, what the reliability of tasks are, and what groups the tasks are appropriate for. Currently, there is little psychometric work on HFCAs, and though some have focused on the ability to capture reliable assessments of means (Aschenbrenner et al., 2025a; Nicosia et al., 2022a; Sliwinski et al., 2018), less work has focused on establishing valid measures that are capable of measuring fluctuations in cognition (Aschenbrenner & Jackson, 2025), leaving many unanswered questions. For example, assuming one can select only a handful of cognitive measures, which tests (or cognitive domains) should be selected? Choice of tests will of course depend on the goals of the research. If one wants to best separate groups based on mean performance (e.g., individuals who are cognitively healthy versus have impairment), a test that shows minimal fluctuation over time may be most desirable. On the other hand, if the goal is to measure fluctuations in accordance with external variables such as sleep or stress, or internal states such as affect or glucose levels, cognitive tests that evince relatively large fluctuations could be preferred. Finally, if the goal is to use variability itself to separate different risk groups (e.g., healthy from cognitively impaired), tests that fluctuate in one group but are stable in another may be needed. It is important to also avoid ceiling and floor effects when selecting tests to utilize, as the constrained time scale of a single HFCA measurement is very brief (a minute or two at most), and the range of scores on any given task may be artificially restricted, substantially biasing estimates of the mean or of variability. Of course, essential questions such as how many assessments should be given and at what times of day are also important to consider.

Unfortunately, due to the aforementioned time constraints imposed on HFCA, there is a dearth of data available to help answer these questions. Moreover, most studies create custom-made cognitive batteries that are tailored to their population of interest and may focus on different constructs or outcomes. For example, many studies aimed at detecting early manifestations of cognitive impairment due to Alzheimer disease may focus on executive function, episodic memory, or processing speed (Aschenbrenner & Jackson, 2023; Baker et al., 2020; Cerino et al., 2021; Nicosia et al., 2022a; Weizenbaum et al., 2024). Researchers studying major depressive disorder may focus on various components of attention (Cormack et al., 2019; Welhaf et al., 2024), and at least one study on insulin resistance focused on working memory ability (Gruber et al., 2024). The point is that all studies, regardless of the population, are by necessity forced to select a few brief measures to implement which likely differ across studies, making it difficult to generate understanding about underlying psychometric properties. Furthermore, many of these batteries run on specialized infrastructure and may be relatively expensive to implement, which may make it difficult for a new investigator to understand which measures are best for their population and to be able to utilize them effectively.

Therefore, we engaged in the current project with several goals in mind. First, we introduce the Cognitive Variability Battery (CVB), a suite of nine cognitive tests that was designed to repeatedly sample multiple cognitive constructs across a relatively wide span of time. Our goal was to develop a broad set of cognitive measures with established psychometric properties and coded in an open-source platform for use by the wider scientific community at virtually no cost. This greatly expands prior work in this area (e.g., Nicosia et al., 2022a; Sliwinski et al., 2018) by tripling the number of cognitive tests that are examined in a single study and expanding the cognitive domains to include measures of attentional control, an aspect of cognition that may change early in neurodegenerative disease (Aschenbrenner et al., 2015; Balota et al., 2010; Twamley et al., 2006). We thoroughly vetted the psychometric properties of our task by calculating an extensive list of properties, including the means, skewness, kurtosis, specific quantiles, between-person reliability (describing the extent to which the outcomes rank-order different people over time), within-person reliability (the extent to which individual participants fluctuate systematically versus randomly), between-person correlations (the degree to which persons who are high on Task A are also high on Task B), within-person correlations (if Task A is high for a participant at a given time point, is Task B also high), and the root mean square of successive differences (capturing the magnitude of change from occasion to occasion across individuals). Thus, researchers can select appropriate tasks based purely on psychometric characteristics, seeking tasks that capture stable between- and within-person effects (i.e., high reliability and high correlations within a domain).

Our second goal was to establish the sensitivity of HFCA testing by examining whether mean estimates and cognitive fluctuations from the nine tasks are related to critical contextual variables, specifically stress, negative affect, and social engagement. Each of these factors have well-established effects on cognitive performance (Calvo & Gutiérrez-García, 2016; Kelly et al., 2017; Padmala et al., 2011; Sandi, 2013; Shields et al., 2016), which will help us establish the sensitivity of our HFCA outcomes (i.e., we should replicate the known findings from the literature). Moreover, we selected these outcomes because they map broadly onto modifiable lifestyle factors that may ultimately reduce the risk of dementia (Livingston et al., 2024) and hence establish the clinical relevance of these outcomes, as they may be targets for eventual behavioral intervention. Once basic validity has been demonstrated, more comprehensive validations, such as comparing to standard neuropsychological batteries, can be explored. Researchers can use our estimates to help design their studies by selecting tasks that evince the largest and most consistent effect sizes.

Lastly, we engage in a series of bootstrapped power analyses to provide recommendations on sample size requirements (in terms of both overall numbers of participants and numbers of repeated assessments) to detect effects of interest, in our case the influence of contextual factors as described above. These analyses will be essential to anyone seeking to implement HFCA testing in their own research programs, by providing guidance on which cognitive tests to select and how many participants/observations may be needed.

## Methods

### Participants

This study was conducted entirely remotely, and therefore individuals were recruited from across the United States using emails to listservs, Facebook postings, and word of mouth. We aimed to recruit a heterogeneous, lifespan sample of participants to be applicable to a wide variety of study designs and therefore enforced minimal inclusion/exclusion criteria. Specifically, the only requirements were that participants be at least 18 years of age and have access to an internet-connected device, preferably a smartphone.

### Materials

**Fig. 1:**
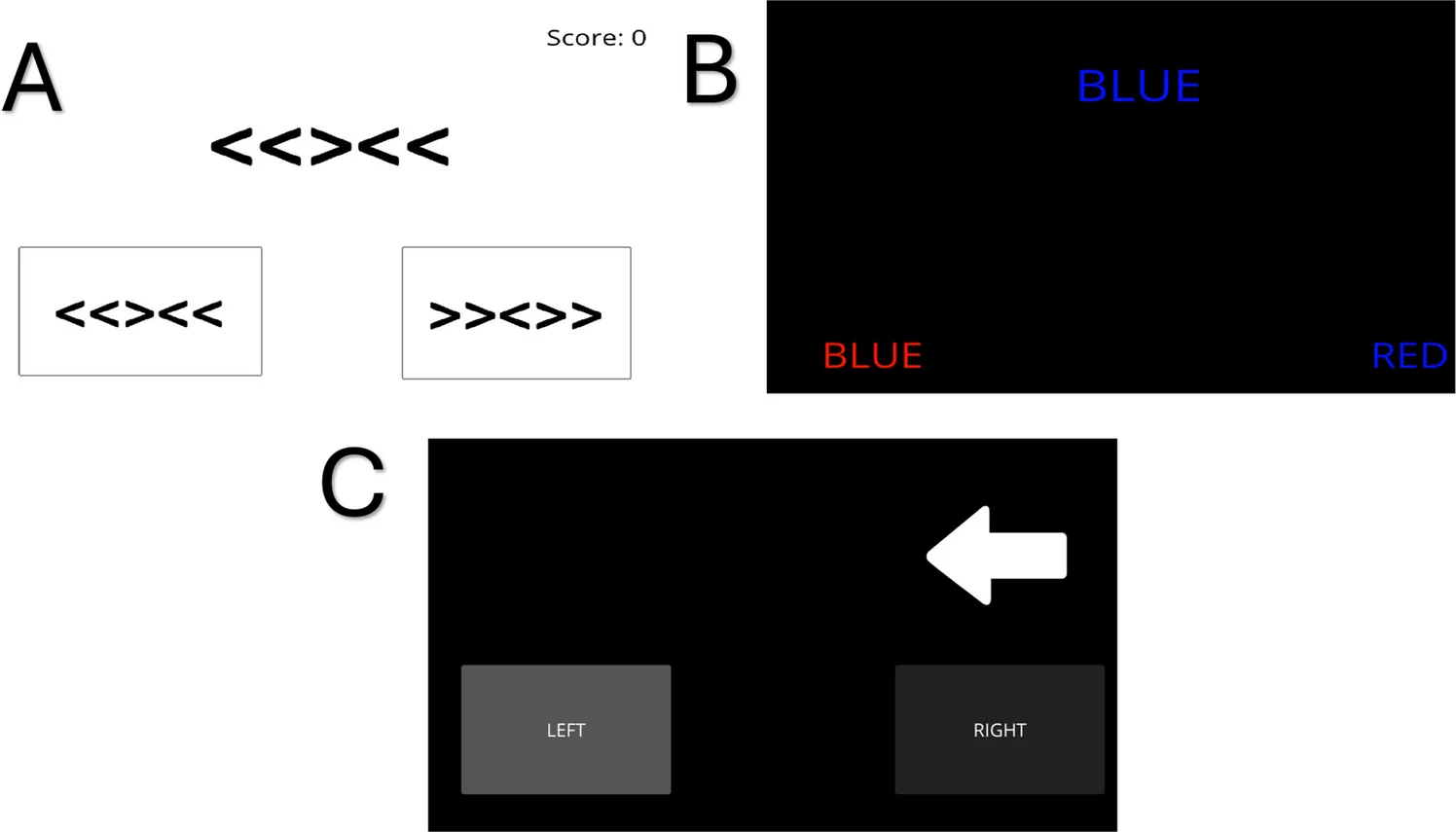
Examples of the three attention tasks. Panel **A** = Flanker squared. Panel **B** = Stroop squared. Panel **C** = Simon squared

**Fig. 2:**
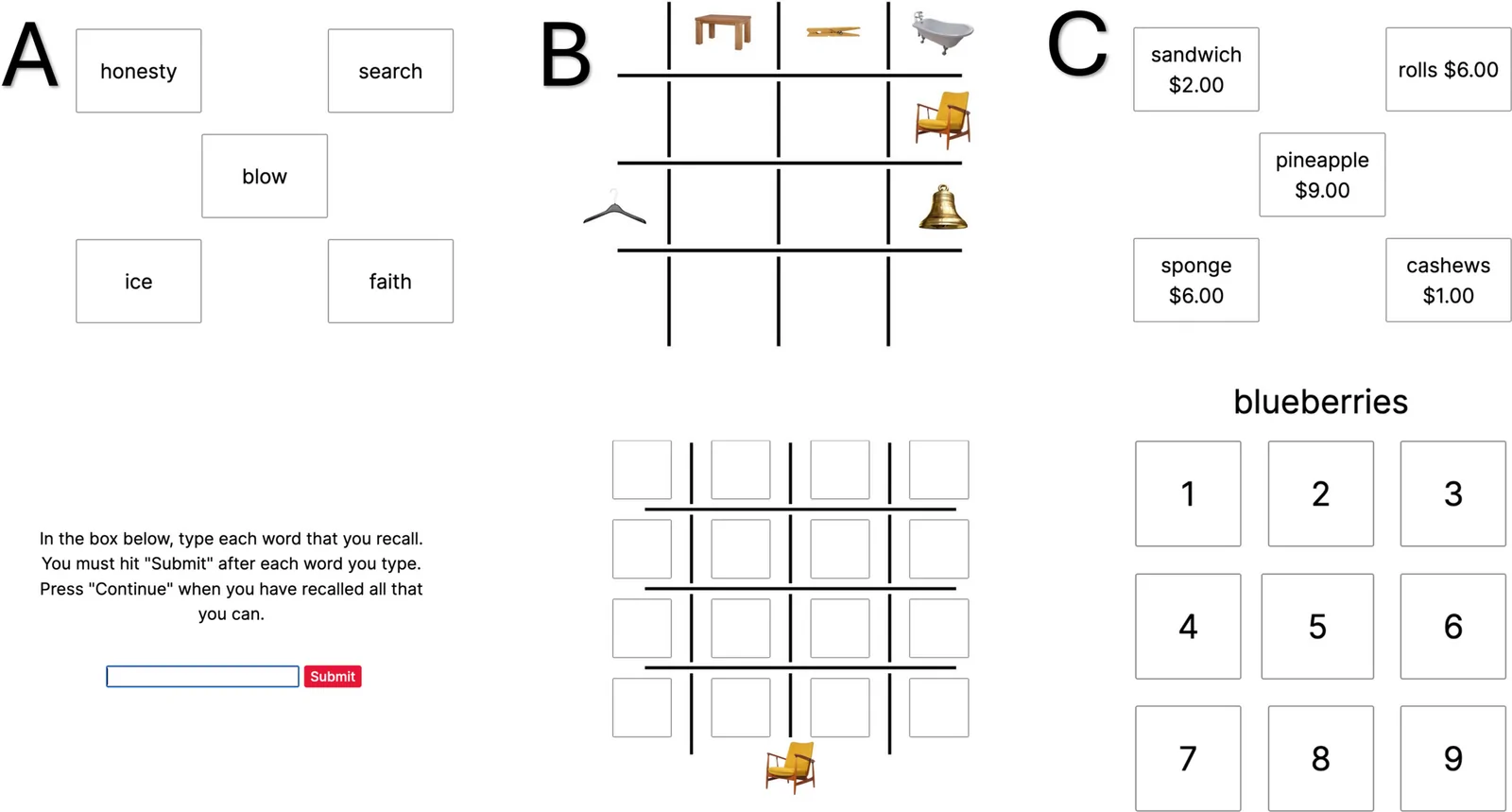
Examples of the three episodic memory tests. Panel **A** = free recall. Panel **B** = spatial memory. Panel **C** = paired associates

**Fig. 3:**
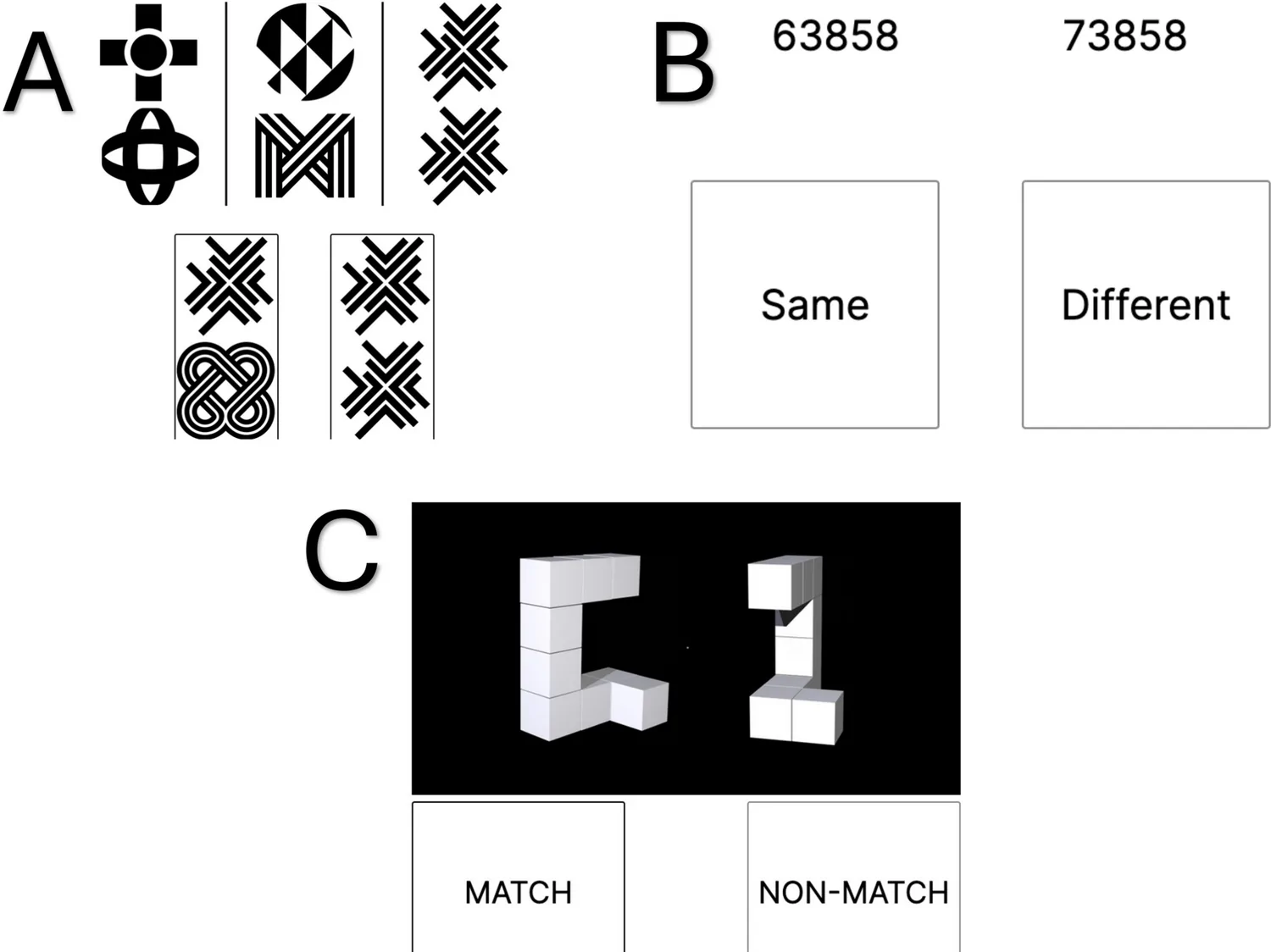
Examples of the three processing speed tests. Panel **A** = symbols. Panel **B** = numbers. Panel **C** = mental rotation

We developed nine cognitive tests that fell into one of three cognitive domains, attentional control, episodic memory, and processing speed. Representative examples of each task are shown in Figs. 1, 2, and 3. The tasks were coded and run on the Gorilla (Anwyl-Irvine et al., 2020) web testing platform and have been submitted to the Gorilla Open Science repository for free and easy access by all members of the scientific community. Participants were also asked multiple questions regarding their current emotional state (stress, positive and negative affect), environment (social interactions), personality traits, and additional cognitive measures such as logical reasoning. To facilitate the use of this dataset by other members of the scientific community, all measures utilized in the study are described below. However, to meet the narrowly prescribed goals of the project, we only examine a subset of these measures. Outcomes that are not utilized in the present analyses are clearly indicated below.

### Attentional control domain

Attentional control is the ability to guide information processing and prioritize specific mental representations in service of particular goals (Burgoyne & Engle, 2020; Oberauer, 2024). Attentional control is particularly important when there are multiple competing stimuli that must be attended to. A common measure of attentional control is the classic Stroop color naming paradigm (MacLeod, 1991; Stroop, 1935), where color words are printed in colored ink, and participants must name the ink color while ignoring the printed text of the word. Despite the clear necessity of attentional control in daily life, its measurement has a rather fraught history (Oberauer, 2024), and such tasks tend to have inadequate psychometric properties for individual differences research (Draheim et al., 2019; Löffler et al., 2024). Therefore, we capitalize on three published tasks of attentional control that have superior characteristics to other tasks of this domain. These tasks are referred to as the “Squared” tasks and were originally developed by Burgoyne et al. (2023). We fully retained all aspects of the original implementation of the tasks and hence only briefly describe each below. Full details can be found in the original publication.

#### Flanker Squared task

Participants are shown an array of arrows as the target stimulus (< <  >  < <), where the central arrow is facing either the same or opposite direction as the outside arrows. In contrast to the standard flanker paradigm, the response options are also arrow stimuli (e.g., <  <  <  <  < and >  >  >  > >). Of the two response options, participants are to select the one where the central arrow matches the direction of the flanking arrows in the target. The target and response options can be manipulated in multiple ways to have varying levels of overlap. We ignore these “conditions” and use as the primary outcome the number of correct responses minus the number of incorrect responses that occurred in 90 s.

#### Stroop Squared task

As in the classic Stroop task, target stimuli are color words printed in colored ink (e.g., RED printed in blue ink), and participants are to determine the ink color of the target and ignore the semantic meaning of the word. The response options are also color words printed in colored ink (e.g., RED printed in red ink and BLUE printed in blue ink). Participants are to select the response option with the semantic meaning (the word) that matches the ink color of the target. As before, we ignore various “conditions” of the stimuli and calculate the number of correct responses minus the number of incorrect responses that occurred in 90 s as the primary outcome.

#### Simon Squared task

As with the traditional Simon task, the target item is an arrow pointing left or right and placed either on the left or right side of the screen. Response options are the words LEFT and RIGHT that appear on either the left- or right-hand side of the screen. Participants must select the response option that matches the direction the arrow is pointing. The primary outcome is the number of correct responses minus the number of incorrect responses that occurred in 90 s, ignoring any condition-level variables.

### Episodic memory domain

#### Free recall task

Participants were shown five words on the screen at a time (one in each corner of the screen and one in the center). They were told they had 15 s to study the items (3 s study time per word). To increase engagement, they were asked to click on the items in the study set that were concrete nouns. After 15 s, a second set of five items was presented followed by a third set for 15 studied items total. Immediately after presentation of the third set of items, participants were prompted to recall as many items as they could by typing answers into a free response box. After recalling as many as they could, they viewed the item list one more time and repeated the immediate recall test (two learning and two test trials). Responses were scored correct if the first three letters of a response matched a studied item. This was implemented to avoid issues with typos and spelling and is an approach that has been used by others (Schmiedek et al., 2010). A new set of 15 items was presented at each testing occasion. Item lists were generated by putting all “target” words from the Semantic Priming Project (Hutchison et al., 2013) and making subsets of 15, ensuring that each list was matched in terms of word frequency and length of each item. The primary outcome was the total number of items correctly recalled summed across both test trials.

#### Paired associates task (pairs)

Five shopping items were shown on the screen (one in each corner and one in the center) together with a single-digit price (e.g., $4.00). Participants had 15 s to encode the item–price pairs. To encourage engagement, they were asked to click ones that would be considered “a good price” for that item. After 15 s, a second set of five items were displayed (for a total of 10 item–price pairs). Immediately after presentation of the second item set, participants were reshown each item in a random order and asked to input the correct price using an on-screen keypad. After testing on each item, the entire sequence was repeated (two learning trials and two test trials). The final outcome was the sum of correct responses across the two trials. This task was designed to be similar to the “Prices” test from the Ambulatory Research in Cognition platform (Nicosia et al., 2022a).

#### Spatial memory task

Images of six common objects (a table, a chair, a bathtub, etc.) were taken from published sources (Jiang et al., 2022) and placed randomly in a 4 × 4 grid. Participants were given 8 s to study the arrangement of items. After 8 s, the grid was cleared, and items were presented one at a time. Participants were instructed to place each item back in the correct location. After the test, items were repeated once more. The outcome was total number of correct placements across both learning trials. This task was designed to be similar to the “Grids” test from the Ambulatory Research in Cognition (ARC) platform.

### Processing speed domain

#### Number comparison

Two separate five-digit strings were presented side by side. Participants were to compare the strings as quickly as possible and determine whether they were the same or different. Each test consisted of 12 sets of items to compare, with half being matches and half different. Items that did not match were “off” by a single digit, and the location of the mismatching digit was controlled such that each position was equally represented across the lists. The primary outcome for this task was the median response time to correct items.

#### Symbols match

Stimuli consisted of vertically stacked pairs of abstract shapes. Three pairs were presented at the top of the screen and two pairs at the bottom. Participants were to select the pair at the bottom that was represented among the three pairs at the top. Twelve trials were presented in total, and the primary outcome was the median response time to correct items. This task was designed to be identical to the “Symbols” task from the ARC platform (Nicosia et al., 2022a).

#### Mental rotation

Two, three-dimensional objects were presented side-by-side. The two objects were either identical or mirror images of each other. All items were rotated by 50, 100, or 150 degrees, and participants were asked to determine whether the items matched or not as quickly as possible. There were 12 trials in each testing session, and the primary outcome was the median response time to correct items. The stimuli were taken from published sources (Ganis & Kievit, 2015).

### Higher-order reasoning domain

Matrix reasoning items were downloaded from the International Cognitive Ability Resource (Condon & Revelle, 2014). Participants viewed a series of five abstract patterns and shapes and were asked to select the sixth item in the sequence from five alternatives. They answered three problems at each testing occasion. This outcome was not examined in the current report.

### Additional daily measures

In addition to the cognitive tasks, participants were also asked about their momentary affect, personality states, and surrounding context at each testing occasion.

#### Personality

Participants were asked single items that map onto the Big Five personality traits (Gosling et al., 2003; John & Srivastava, 1999). Specifically, they were prompted with the phrase “In the LAST HOUR, I see myself as …” and then shown the following items: (1) extraverted, enthusiastic, (2) agreeable, sympathetic, (3) dependable, self-disciplined, (4) calm, emotionally stable, (5) open to new experiences, complex. Participants input their response to each item using a five-item Likert scale (disagree strongly, disagree a little, neutral, agree, agree strongly). Personality was not examined in the current report.

#### Affect and stress

Participants were prompted with the phrase “I am feeling ____ about my life right now” and shown three items: (1) positive, (2) negative, (3) stressful. They input their responses on a five-point Likert scale (very slightly/not at all, a little, moderate, quite a bit, extremely).

#### Context/situation

Participants were shown seven different situations and were asked to select any that applied to them over the past hour. The situations were as follows: (1) was late for something, (2) interacted with people, (3) was relaxing, (4) was working, (5) was fatigued, (6) something pleasant happened, and (7) was familiar with the circumstances. Only the “interacted with people” variable was examined in the current report.

#### Study procedure

Potential participants were engaged via flyers and postings on various social media platforms. If interested in the study, they navigated to an intake screen implemented in the RedCAP (Harris et al., 2019) electronic data capture system hosted at Washington University in St. Louis, where they read the informed consent document and answered basic demographic questions (age, sex, etc.). Upon completion of this survey, participants were scheduled for a remote baseline visit with a member of the study team conducted via Microsoft Teams or Zoom. During this session, the team member demonstrated each of the cognitive tasks and answered any general questions the participant had. After the baseline session was completed, participants were automatically enrolled in the HFCA portion of the study.

Participants were scheduled to receive test notifications three times per day (at approximately 9:00 AM, 1:00 PM, and 6:30 PM Central Time). Notifications were delivered via SMS text message through the RedCAP system which contained a message saying “Your testing session is now available” with a custom link that directed them to the Gorilla platform to complete the testing. Data were sent to Gorilla using standard TLS and stored encrypted at rest using industry standards. No personal identifying information was sent to Gorilla (i.e., phone numbers are not shared). Each testing session consisted of the personality, affect, and context questions followed by six cognitive tasks (two from each domain) and finished with the logical reasoning test. The six cognitive tests that were administered were rotated across sessions such that each pair of tests occurred equally often with each of the other pairs. A total of 90 notifications were sent (three per day for 30 days), and thus each cognitive test was administered a total of 60 times. If participants missed one or more of the sessions, they were allowed to retake it at the end of the 30 days. Participants were rewarded with tiered reimbursement based on the number of tests completed and bonus payments for high adherence. Some individuals also chose to complete portions of the testing for research credits. The total possible compensation was $215. We did not set an a priori sample size and instead conducted the study for 1 year from summer of 2024 to summer of 2025 in accordance with availability of study funding. Our initial goal was to obtain data on at least 100 individuals.

#### Statistical analysis

All analyses were conducted using R version 4.5.2 (R Core Team, 2021). Data manipulation was facilitated by dplyr (Wickham et al., 2014), basic summary statistics such as means and correlations were calculated using base R functions, skew and kurtosis values were calculated using the psych package (William Revelle, 2025), and intraclass correlations (ICCs) and contextual effects including power analyses were conducted using lme4 (Bates et al., 2015). We organize our analyses around three central questions, each of which applies to two aspects of our data: mean performance and variability. Cognitive variability was defined as the root mean square of successive differences (RMSSD; von Neumann et al., 1941).

Analysis 1: Expanding upon the analyses by Aschenbrenner and Jackson (2025), our first goal was to investigate the psychometric properties of the tests in the CVB. Specifically, we calculate means, standard deviations, skewness, kurtosis, and distributions for the mean and RMSSD in each task and provide estimates of reliability using ICC, defined as the proportion of between-person variance relative to the total variability. This ICC statistic can be interpreted as the expected correlation between any two points in the time series. We calculated the ICC on individual assessments (single tests) as well as averages of each test across a certain time frame (specifically, 5, 10, 15 and 20 assessments). This is meant to capture the increasing reliability of each outcome as a function of the number of tests administered. In addition to the between-person reliability, we also report a metric of within-person reliability which quantifies the extent to which occasion-to-occasion fluctuations can be attributed to systematic versus random sources. While there are several well-established techniques to do this (Schmiedek et al., 2013; Sliwinski et al., 2018), they all rely on having access to single trial scores which are not readily applicable to our tasks (particularly the free recall test). We therefore calculated the within-person split-half reliability, defined as the correlation between the first half of the test and the second half (e.g., for the attentional control tasks, we calculated correct minus incorrect scores for each 45-s epoch of testing; for the memory tests we compared accuracy after the first learning trial and the second learning trial), across all days of assessments. The resultant correlations were then averaged across participants. As these correlations are based on only half of each task, they likely underestimate the true correlation, and therefore we also apply a Spearman–Brown correction [(2 × *r*)/1 + *r*)] to estimate the reliability of the full length of the task. Of course, this correction assumes the two halves of each task are independent, which may not equally apply to all measures, particularly the memory tests, and should be interpreted cautiously. By presenting both raw and corrected correlations, we provide a range of plausible correlation values with the understanding that neither estimate can be taken as an unbiased estimate of the true within-person reliability. We report the correlations between each of the nine cognitive tests at baseline as well as the correlation among the person averages (i.e., the average score for each outcome across all assessments). Finally, we report the average within-person correlation for each pair of tasks, specifically, for each individual, we correlated the scores for two tasks at a time and then averaged that correlation across the entire 139 participants in the sample.

*Analysis 2*: The aim of the second set of analyses was to examine the relationship between each cognitive outcome (again, both mean scores and variability) and the key contextual factors (i.e., stress, negative affect, and social interactions). To separate the influence of between- and within-person effects, each contextual variable was centered around each individual’s mean (termed the “WP effect,” which reflects the influence of a deviation from the individual’s typical level of performance). The person-level mean was also included to assess between-person effects (the “BP effect”). A series of multilevel regression models were constructed for each cognitive test and each daily predictor (stress, negative affect, social interactions). Scores on the cognitive tests were the outcome with each daily predictor entered in separate models. Age was included as a covariate along with a random intercept and random slopes of the WP effect across participants. A total of 27 separate models were fit (nine tests by three daily predictors), and there was no adjustment for multiple comparisons. We minimize concerns regarding multiple comparisons by (a) focusing on effect size estimation rather than a strict “significant versus not” decision, (b) reporting all model outcomes, not just ones that reach significance or look interesting, and (c) interpreting effects holistically, that is, looking for patterns of effects within the domain rather than interpreting isolated effects. Accordingly, we avoid interpreting individual effects as “present” or “absent” based on statistical thresholds. For the analyses of the RMSSD metric, a within-person effect of context is not defined; therefore, we collapse across occasions and examine a single RMSSD metric for each person and focus only on the between-person context effect using single-level linear regression.

*Analysis 3*: The goal of Analysis 3 was to determine the optimal assessment strategy in terms of numbers of participants to be recruited as well as the number of assessments needed to detect effects of interest. Therefore, we conducted a bootstrapped analysis of our data where we specified varying numbers of participants (from 50 to 300 in 50-participant increments) and number of repeated assessments (3, 10, 25, 50, 75, or 100). For each combination of parameters, we sampled with replacement from our observed data and used linear mixed effects modeling to extract parameter estimates of each between-person and within-person effect. For example, for the parameter combination of 50 participants and three assessments, we randomly selected 50 participants (with replacement) from our overall sample, and within those 50 individuals, we randomly selected three assessments (with replacement). We then calculated the between-person (BP) and within-person (WP) effects as defined above and ran a linear mixed effects model to extract estimated effects. This was repeated 250 times for each parameter combination. We defined power as the proportion of the 250 models that were statistically significant (95% confidence intervals excluded zero). This was repeated for each of our nine cognitive tests. The process was repeated for the RMSSD, but again, a WP effect is not defined, so we examine only the BP effects using single-level regression.

## Results

Due to the understanding that within-person effects are small and difficult to detect (Wright et al., 2025), we adopted a minimum criterion of 45 completed sessions to be included for subsequent analyses. A total of 139 participants met this criterion after a 1-year recruitment period. The average age of this sample was 37.5 (*SD* = 17.8, range = 18–79), and the average number of sessions completed was 81.6 (*SD* = 12.4, range = 45–90). In terms of self-reported gender and race/ethnicity, 76% of the sample reported being female, 51% as White/Caucasian, 24% Black/African American, 24% Asian, < 1% Native American, 3% “Other,” and 6% Hispanic or Latino. The sample reported relatively low stress (mean = 2.2, *SD* = 0.92, possible scores = 1–5), low negative affect (mean = 1.9, *SD* = 0.8, possible scores = 1–5), and somewhat frequent social activity (mean = 0.6, *SD* = 0.3, possible scores = 0–1). Of the 11,345 individual sessions collected in this study, 90.1% were completed on a mobile device (i.e., a smartphone), 9.7% were completed on a desktop or laptop computer, and < 1% were completed on some other device (e.g., an iPad or other tablet computer).

*Analysis 1:* The psychometric properties of mean performance on each cognitive task are summarized in Table 1, and of the RMSSD in Table 2. First considering Table 1, examination of the means and standard deviations of each task indicates some ordering of difficulty within each domain. For example, Flanker and Stroop had very similar scores, whereas Simon was noticeably easier (had a higher score). When converted to a percentage of the total possible scores, free recall was clearly the most difficult (45% correct), followed by pairs (59%) and then spatial memory (70%). Finally, number comparison was clearly the fastest processing speed test, followed by symbols and then mental rotation.

**Table 1:** Psychometric characteristics of mean scores for each test in the CVB battery

Task	Mean (*SD*)	Skew	Kurtosis	0.1	0.3	0.5	0.7	0.9	ICC	ICC_5_	ICC_10_	ICC_15_	ICC_20_	WP Rel
Flanker	34.7 (17.5)	− 0.40	− 0.98	7.9	24.7	37.9	47.5	53.7	0.72	0.83	0.85	0.86	0.87	0.55 (0.67)
Stroop	38.9 (15.8)	− 0.52	− 0.43	15.2	32.4	40.2	48.3	57.7	0.69	0.82	0.84	0.85	0.86	0.54 (0.67)
Simon	51.4 (15.4)	− 0.99	0.72	29.6	46.6	55.0	61.3	67.1	0.64	0.79	0.81	0.81	0.82	0.51 (0.64)
Free recall	13.4 (6.3)	0.42	− 0.65	6.5	9.5	12	16	22.4	0.70	0.85	0.88	0.89	0.90	0.58 (0.71)
Spatial	8.3 (2.3)	− 0.60	− 0.24	5.3	7.2	8.6	10.1	11.0	0.42	0.72	0.79	0.81	0.81	0.46 (0.60)
Pairs	11.7 (4.0)	− 0.25	− 0.85	6.0	9.4	11.9	14.5	16.5	0.57	0.81	0.84	0.87	0.87	0.57 (0.71)
Symbols	1,654 (590)	0.67	0.54	1056	1340	1552	1825	2522	0.61	0.78	0.81	0.83	0.84	0.12 (0.17)
Numbers	1,425 (530)	0.61	0.02	911	1115	1276	1589	2233	0.67	0.85	0.88	0.89	0.90	0.12 (0.17)
Mental rotation	1,819 (907)	1.90	6.9	1014	1354	1628	2045	2832	0.60	0.74	0.78	0.79	0.79	0.16 (0.23)

Flanker, Stroop, and Simon scores reflect the number of correct responses minus the number of incorrect responses (higher is better). Free recall, spatial, and pairs reflect the sum of correctly recalled items across two learning trials (max scores = 30, 12, and 20, respectively). Symbols, numbers, and mental rotation scores are in milliseconds and reflect the median response time of correct items. Variables 0.1, 0.3, 0.5, 0.7, and 0.9 reflect the 10th, 30th, etc., percentile of each task. ICC = intraclass correlation and serves as a measure of reliability. ICC_5_ is the ICC of five tests averaged together, and then 10 tests, and so on. WP Rel = the average correlation between the first and second half of each test, averaged across days and then across participants. Spearman–Brown-corrected version presented in paratheses

**Table 2:** Psychometric characteristics of RMSSD for each test in the CVB battery

Task	Mean (*SD*)	Relative RMSSD	Skew	Kurtosis	0.1	0.3	0.5	0.7	0.9	ICC_5_	ICC_10_	ICC_15_	ICC_20_
Flanker	11.0 (5.3)	32%	0.93	0.37	5.32	7.49	9.69	13.2	18.0	0.38	0.48	0.53	0.60
Stroop	11.2 (4.9)	29%	0.79	0.16	5.87	7.95	9.93	13.7	17.9	0.29	0.42	0.52	0.55
Simon	12.2 (6.46)	24%	0.70	− 0.33	5.07	7.85	11.1	15.2	21.2	0.38	0.49	0.56	0.60
Free recall	4.9 (1.8)	37%	0.33	1.27	2.99	4.16	4.68	5.44	7.35	0.28	0.41	0.51	0.49
Spatial	1.5 (1.1)	18%	− 0.38	− 0.30	1.79	2.83	3.51	4.01	4.69	0.30	0.44	0.49	0.57
Pairs	4.3 (1.0)	37%	0.11	− 0.05	3.08	3.72	4.23	4.82	5.64	0.16	0.25	0.33	0.38
Symbols	503 (284)	30%	1.1	0.94	196	319	441	599	907	0.34	0.45	0.54	0.60
Numbers	406 (281)	28%	1.7	3.86	148	210	339	481	763	0.36	0.50	0.53	0.66
Mental rotation	698 (436)	38%	1.6	3.02	287	432	582	778	1288	0.37	0.51	0.54	0.56

It is not possible to calculate an ICC of a single assessment as done in Table 1, as the RMSSD metric by definition does not exist for a single score, and we cannot calculate a WP reliability metric as it also requires trial-level information

More important than raw means, however, is the fact that most tests were relatively symmetrical and normal-tailed, as reflected by the skew and kurtosis values. Most skew values were less than an absolute value of 0.75, indicating, at most, slight levels of skew according to common rules of thumb (Blanca et al., 2013). The exceptions were the Simon task, which was “moderately” negatively skewed, and the mental rotation task, which exhibited extreme positive skew. In terms of kurtosis, the results were much the same, with exceptions for the Flanker and pairs tasks, which were noticeably platykurtic, and the mental rotation task which was extremely leptokurtic. The quantiles of each score complement these results and additionally indicate that the tests appear devoid of a floor effect, as the 0.1 quantile clusters away from zero. Neither the attention tests nor the processing speed tests have a ceiling (scores could range up to infinity); however, we did appear to avoid a ceiling effect in the memory tests as well (based on the scores at the 0.9 quantile).

Finally, and as expected, the ICCs of a single administration of each task were rather poor (the lowest being 0.42 for spatial memory). Interestingly, the ICCs for Flanker and free recall, while only 0.70, are on par with more detailed and comprehensive neuropsychological tests that are routinely administered such as the Symbol Search task (test–retest *r* = 0.74), the California Verbal Learning Test short delay recall (*r* = 0.65) and long delay recall (*r* = 0.74), and Trail Making part A (*r* = 0.66), to name a few (Calamia et al., 2013). The ICCs increased dramatically when averaging together just five tests and continued to increase with 10 and 15 tests but appeared to max out at 15 tests with ICC values near 0.90. Therefore, continued gains in reliability of the mean estimate with more administrations are likely to be minimal. The within-person reliability of the attention tests and memory tests ranged from 0.46 to 0.58, values that are in line with those reported elsewhere (Sliwinski et al., 2018). The within-person reliability for the processing speed tests was surprisingly low, suggesting fluctuations in processing speed were dominated by trial-level noise.

The same metrics were calculated for the RMSSD metric and are presented in Table 2. As the raw values of RMSSD are difficult to interpret in isolation, we also present a “relative RMSSD” metric which is simply the RMSSD score divided by the task mean (from Table 1). With one exception for the spatial memory task, the RMSSD values were 25–40% of the mean: e.g., from one trial to the next, flanker scores typically changed by ~ 11 units (32% of 34.7). The ICCs of the RMSSD were much lower than the mean, and even 20 assessments resulted in subpar reliability estimates.

Turning to the correlations across the different tasks, the inter-correlations of the mean performance at the first assessment of each task as well as the average of all responses across the entire study are presented in Table 3. Looking first at the bottom diagonal, which reflects the correlations among the initial administration only, the tests within each domain correlate only modestly, with average correlations hovering around 0.5. Correlations across tests from different domains were of roughly that same magnitude, with few exceptions. This might suggest that a single administration of various remote cognitive tests taps a single underlying latent factor rather than capturing unique cognitive domains. Along the top diagonal, reflecting correlations among the task averages, the correlations among the attention tests and the memory tests increased substantially (~ 0.8), but surprisingly, the correlations among the processing speed tasks were relatively unchanged, at least when concerning the mental rotation task.

**Table 3:** Correlations between the first assessment of each task (bottom diagonal) and the average of each test across the entire study (top diagonal)

	Flanker	Stroop	Simon	FR	Spatial	Pairs	Symbols	Number	Mental
Flanker	1	0.86	0.81	0.61	0.74	0.70	− 0.37	− 0.38	0.08
Stroop	0.59	1	0.86	0.62	0.70	0.70	− 0.22	− 0.26	0.13
Simon	0.48	0.55	1	0.53	0.61	0.59	− 0.07	− 0.08	0.23
FR	0.5	0.49	0.49	1	0.71	0.71	− 0.23	− 0.30	0.12
Spatial	0.42	0.37	0.46	0.44	1	0.79	− 0.38	− 0.37	0.02
Pairs	0.42	0.41	0.46	0.56	0.56	1	− 0.19	− 0.19	0.15
Symbols	− 0.49	− 0.49	− 0.52	− 0.45	− 0.48	− 0.49	1	0.88	0.45
Number	− 0.53	− 0.43	− 0.54	− 0.58	− 0.44	− 0.51	0.69	1	0.43
Mental	− 0.17	− 0.12	− 0.15	− 0.14	− 0.23	− 0.06	0.37	0.34	1

FR = free recall

The within-person correlations (i.e., on days when you are high on one task, are you high on another) are presented in Table 4. As shown, cross-domain correlations were small and rarely exceeded 0.2 in magnitude. Thus, it is not the case that individuals are “generally bad” on all tests on one day versus another. The correlations for the attentional control tasks were moderate at around 0.40, smaller for the memory tests at ~ 0.25 and ~ 0.30 for processing speed. Table 5 lists the across-task correlations of the RMSSD (does high RMSSD on one task correspond to similarly high RMSSD on another task). As shown, the estimates vary widely across tasks, with the attention tasks exhibiting rather high correlation values approaching 0.80, demonstrating strong between-task consistency among successive, trial-to-trial fluctuations. The number comparisons task and symbols task were also highly correlated at 0.70. The remaining within- and across-domain correlations were relatively modest.

**Table 4:** Average within-person correlations for each of the nine cognitive tasks in CVB

	Flanker	Stroop	Simon	FR	Spatial	Pairs	Symbols	Number	Mental
Flanker	1								
Stroop	0.46	1							
Simon	0.39	0.37	1						
FR	0.17	0.20	0.21	1					
Spatial	0.18	0.16	0.21	0.23	1				
Pairs	0.14	0.21	0.20	0.27	0.20	1			
Symbols	− 0.22	− 0.24	− 0.17	− 0.08	− 0.09	− 0.04	1		
Number	− 0.16	− 0.17	− 0.13	− 0.05	− 0.05	− 0.03	0.24	1	
Mental	− 0.24	− 0.17	− 0.06	0.05	0.01	0.11	0.32	.28	1

The number of assessments for each pairwise correlation will vary slightly based on the number of days the participant completed. FR = free recall

**Table 5:** Across-task correlations for the RMSSD metric for each of the nine tasks in CVB

	Flanker	Stroop	Simon	FR	Spatial	Pairs	Symbols	Number	Mental
Flanker	1								
Stroop	0.77	1							
Simon	0.78	0.77	1						
FR	0.19	0.25	0.23	1					
Spatial	0.40	0.42	0.44	0.10	1				
Pairs	0.32	0.40	0.41	0.28	0.54	1			
Symbols	0.31	0.44	0.45	0.13	0.39	0.42	1		
Number	0.24	0.32	0.40	− 0.03	0.38	0.29	0.70	1	
Mental	0.10	0.14	0.21	0.08	0.33	0.15	0.55	0.47	1

FR = free recall

*Analysis 2:* The sensitivity of each task to contextual effects is summarized in Table 6, with an emphasis on the magnitude and consistency of effects across measures. All three attention tasks exhibited consistent effects across all three contextual variables. Specifically, higher stress, higher negative affect, and less frequent social interactions were all associated with reduced attention performance. Associations with memory were slightly more task-specific. For example, while all three memory tasks were associated with BP differences in negative affect (higher negative affect = lower memory ability), associations with stress were larger and more consistent for free recall and spatial memory, whereas the effect for pairs was smaller and less consistent. The opposite pattern was true for social interactions, where the most consistent effects appeared in spatial memory and pairs, whereas the effect on free recall was inconsistent. In the absence of a specific hypothesis as to why stress would *not* affect paired associates or why social interactions would *not* manifest on free recall, it seems prudent to conclude that spatial memory is simply a more sensitive or perhaps more psychometrically sound cognitive test, in that it consistently reveals these between-person differences. In terms of processing speed, symbols and number comparison were consistently associated with stress and negative affect in the expected directions, whereas no task showed a consistent relationship with social interactions.

**Table 6:** Parameter estimates and 95% confidence intervals from a linear mixed-effects model estimating the association between each CVB task and BP or WP effects of key contextual variables

	Stress		Negative affect		Social interactions	
Task	BP	WP	BP	WP	BP	WP
Flanker	** − 4.0 [− 7.6, − 0.5]**	− 0.51 [− 1.03, 0.01]	** − 8.21 [− 12.3, − 4.2]**	− 0.55 [− 1.11, 0.01]	**20.0 [9.2, 30.9]**	− 0.55 [− 1.28, 0.17]
Stroop	** − 4.5 [− 7.8, − 1.3]**	− 0.3 [− 0.75, 0.23]	** − 7.9 [− 11.6, − 4.2]**	** − 0.6 [− 1.12, − 0.03]**	**16.6 [6.6, 26.7]**	− 0.6 [− 1.28, 0.02]
Simon	** − 3.5 [− 6.7, − 0.3]**	− 0.04 [− 0.5,0.5]	** − 5.5 [− 9.2, − 1.9]**	** − 0.9 [− 1.5, − 0.25]**	**19.7 [10.2,29.2]**	0.04 [− 0.8, 0.8]
Free recall	** − 1.32 [− 2.6, − 0.02]**	** − 0.2 [− 0.37, − 0.04]**	** − 1.94 [− 3.44, − 0.45]**	** − 0.40 [− 0.59, − 0.20]**	2.52 [− 1.55, 6.6]	− 0.26 [− 0.55, 0.03]
Spatial	** − 0.56 [− 1.02, − 0.09]**	− 0.02 [− 0.12, 0.09]	** − 1.09 [− 1.6, − 0.6]**	− 0.1 [− 0.22, 0.01]	**2.32 [0.91, 3.74]**	** − 0.27 [− 0.46, − 0.08]**
Pairs	− 0.54 [− 1.39, 0.30]	− 0.03 [− 0.17, 0.11]	** − 1.24 [− 2.19, − 0.28]**	** − 0.21 [− 0.36, − 0.06]**	**2.76 [0.19, 5.34]**	** − 0.24 [− 0.43, − 0.04]**
Symbols	**154.72 [57.7, 252]**	7.26 [− 13.4, 27.9]	**223.47 [113, 334]**	− 14 [− 41, 13]	− 262 [− 577, 53]	**38 [7, 69]**
Number	**140 [57 − 223]**	0.4 [− 13, 14]	**211 [117, 306]**	− 13 [− 28, 3]	− 188 [− 459, 83]	**44 [19, 69]**
Mental	58 [− 103, 219]	18 [− 14, 50]	− 78 [− 260, 105]	** − 56 [− 97, − 16]**	− 44 [− 559, 471]	**71 [24, 117]**

Items in bold have 95% CIs that exclude zero, but interpretation of effects does not rely on the notion of statistical significance

Next, we examined the within-person associations. Perhaps not surprisingly, within-person associations were much more sporadic. In terms of attention, both Stroop and Simon were associated with fluctuations in negative affect such that when negative affect is unusually high, attention performance is correspondingly low. Similarly, two memory tests (free recall and pairs) were associated with WP negative affect. Free recall was also associated with WP stress, and spatial memory was related to WP social interactions. Finally, all three processing speed tests were related to WP variation in social interactions, such that a recent social interaction was related to *slower* processing speed. Nevertheless, it is key to note that all measures evinced at least one effect of nontrivial magnitude, establishing the predictive validity of these measures.

The analogous (but reduced, i.e., using single-level regression to evaluate BP effects but losing the ability to examine within person effects) analyses for RMSSD are presented in Table 7. As shown, for the attention and memory tests, all effects were small and centered near zero (with two noted exceptions, spatial memory with negative affect and pairs with social interactions). In contrast, the RMSSD for each of the processing speed tasks was consistently associated with all three contextual variables, with the sole exception of social interactions on mental rotation speed.

**Table 7:** Parameter estimates and 95% confidence intervals from a single-level regression model estimating the association between RMSSD in each CVB task and BP effects key contextual variables

Task	Stress	Negative affect	Social interactions
Flanker	− 0.64 [− 1.64, 0.37]	− 0.28 [− 1.45, 0.90]	− 0.90 [− 4.08, 2.29]
Stroop	0.20 [− 0.74, 1.14]	0.78 [− 0.31, 1.86]	− 2.15 [− 5.1, 0.81]
Simon	0.43 [− 0.80, 1.66]	1.00 [− 0.42, 2.42]	− 3.5 [− 7.34, 0.34]
Free recall	− 0.26 [− 0.63, 0.11]	− 0.39 [− 0.81, 0.04]	0.39 [− 0.78, 1.56]
Spatial	0.23 [− 0.01, 0.46]	**0.35** **[0.08, 0.62]**	− 0.71 [− 1.45, 0.03]
Pairs	0.07 [− 0.16, 0.29]	0.10 [− 0.16, 0.36]	** − 0.70** **[− 1.40, − 0.01]**
Symbols	**86** **[26, 146]**	**145** **[77, 213]**	** − 369** **[− 553, − 184]**
Number	**101** **[42, 159]**	**162** **[97, 228]**	** − 208** **[− 397, − 18]**
Mental	**107** **[17, 196]**	**149** **[46, 252]**	− 259 [− 544, 27]

Items in bold have 95% CIs that exclude zero, but interpretation of effects does not rely on the notion of statistical significance

**Fig. 4:**
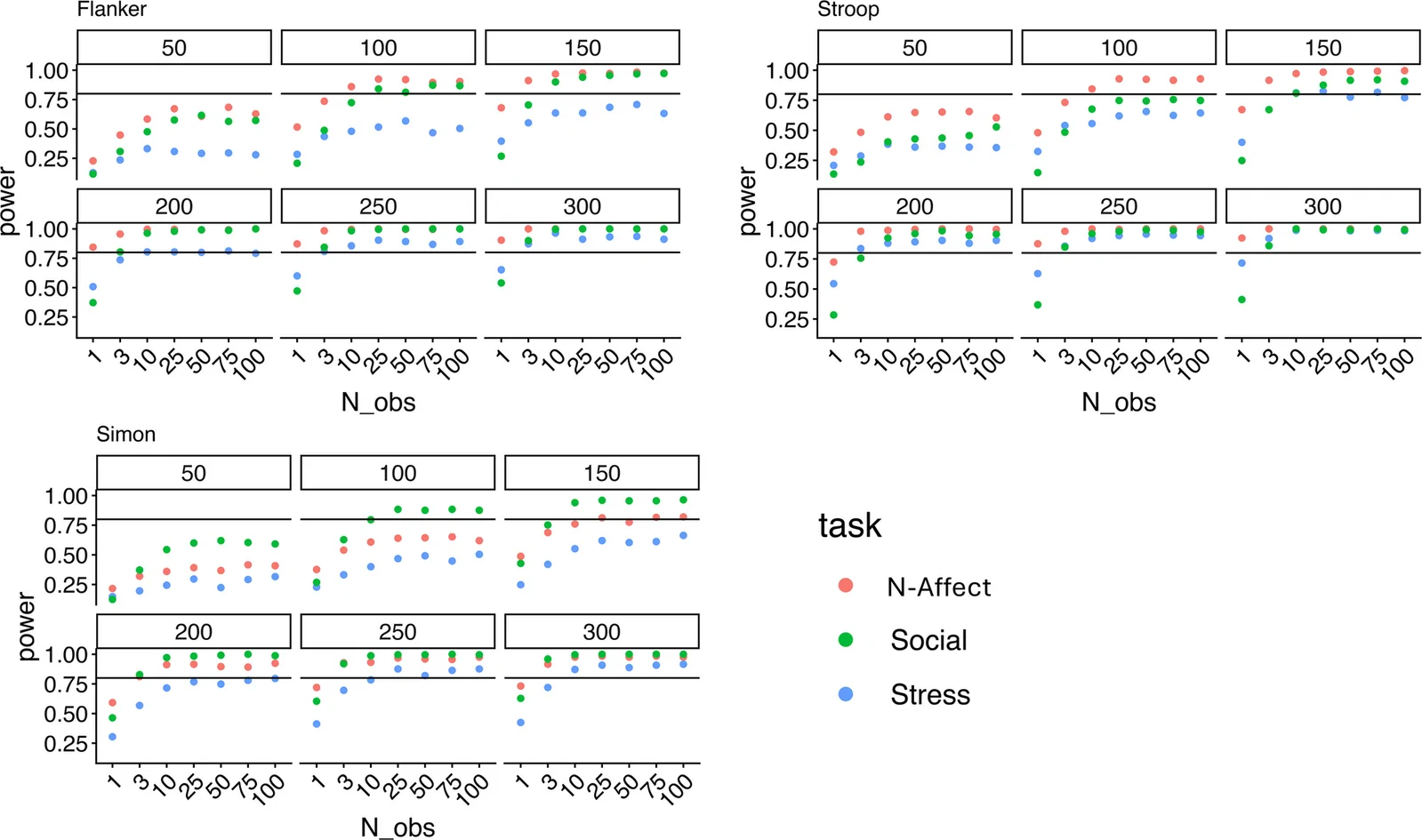
Power curves of the attention tests to detect between-person effects of each contextual variable (negative affect, social interaction, perceived stress)

**Fig. 5:**
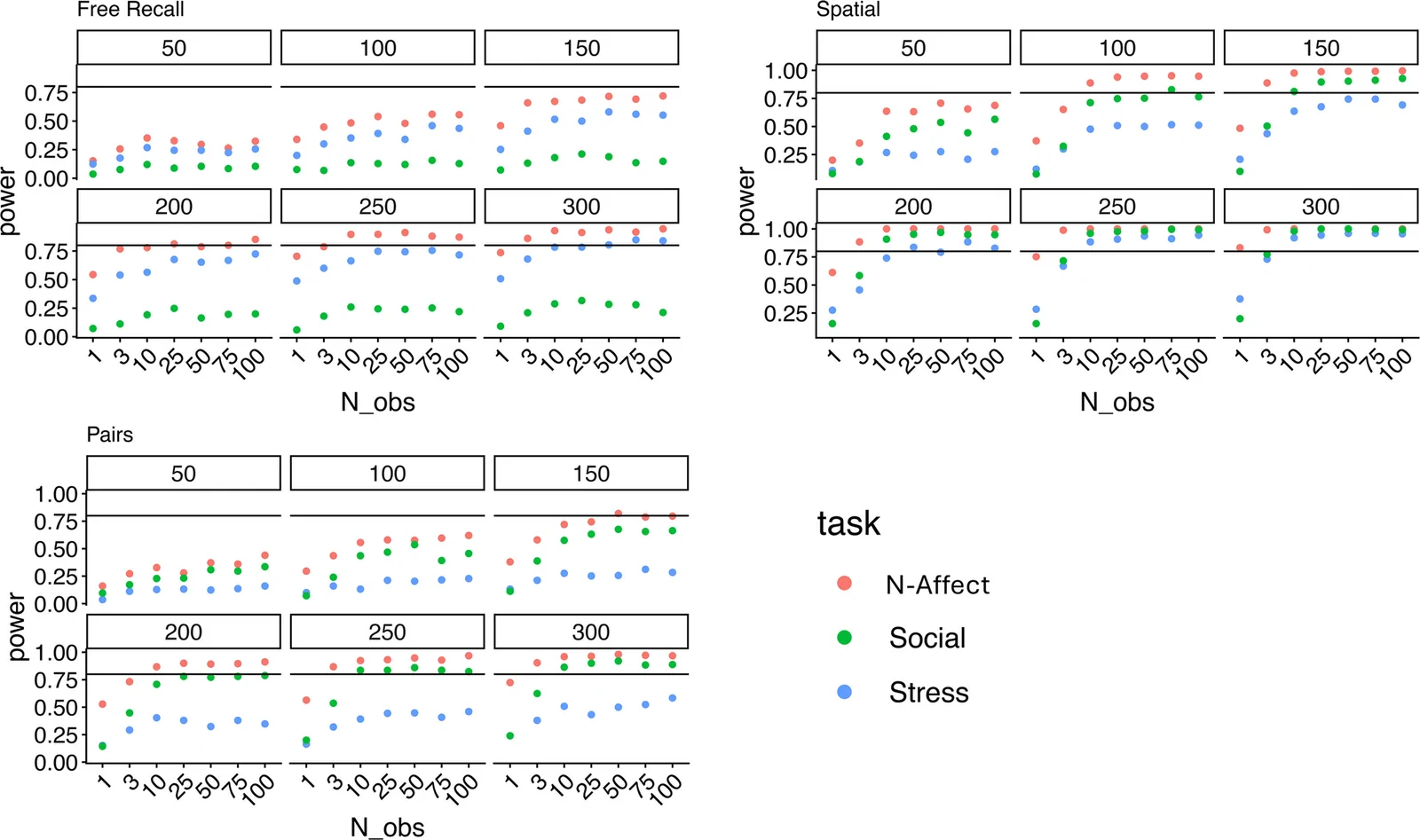
Power curves of the episodic memory tests to detect between-person effects of each contextual variable (negative affect, social interaction, perceived stress)

**Fig. 6:**
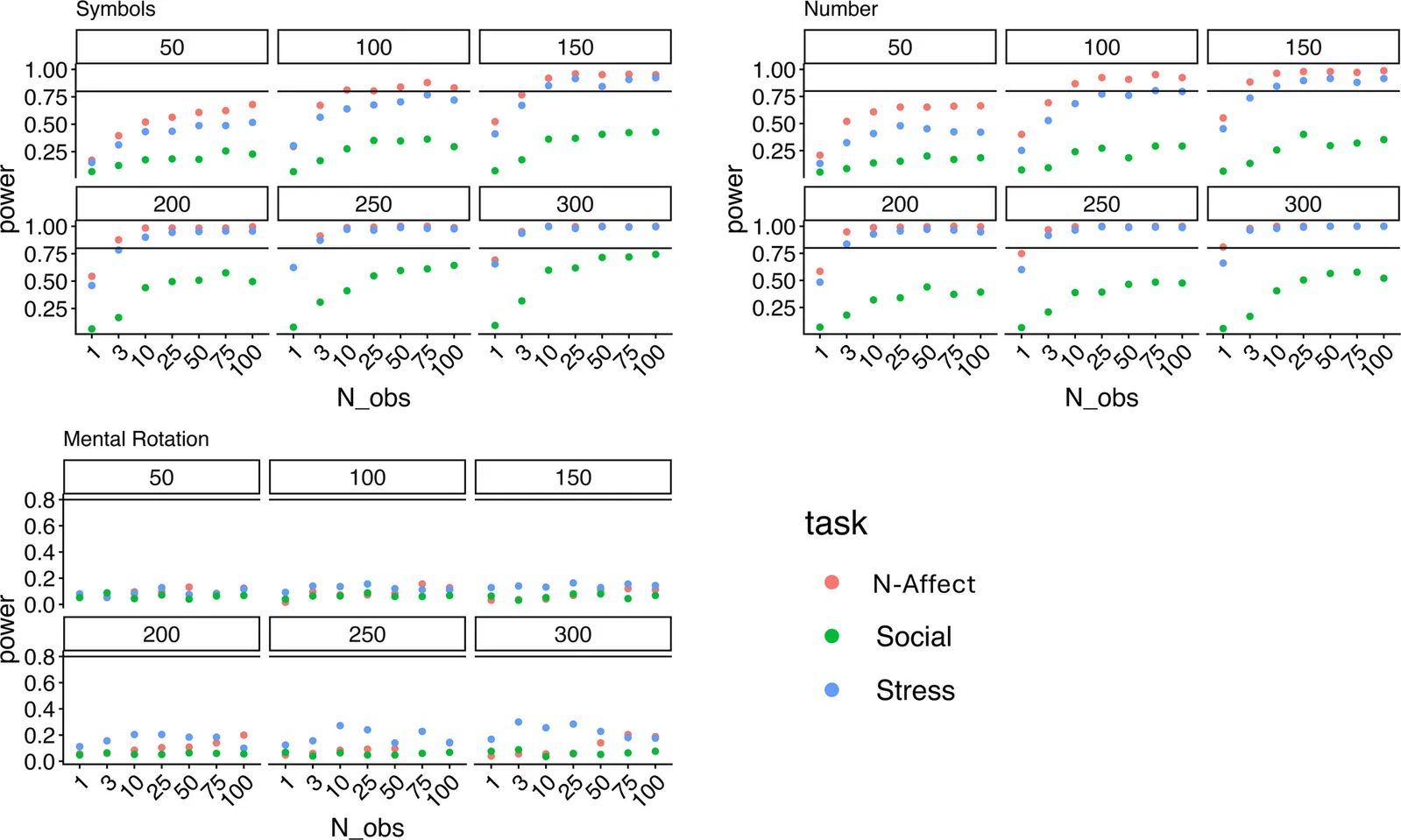
Power curves of the processing speed tests to detect between-person effects of each contextual variable (negative affect, social interaction, perceived stress)

*Analysis 3:* The results of the bootstrapped power analysis for the means are presented in Figs. 4, 5, and 6 for attention, memory, and processing speed, respectively. Each figure plots observed power as a function of the number of simulated assessments (*x*-axis) and number of participants (different facets) to detect a BP effect of negative affect, social interactions, or stress (different colored points). We consider each of these figures in turn, but in general, it is clear that the ability to obtain sufficient power to detect BP effects will vary greatly based on the nature of the cognitive task as well as the contextual variable under consideration.

First, in terms of attention, all three tests would be adequately powered to detect BP differences in social interactions with 100–150 participants and between 10 and 25 repeated assessments. Both Flanker and Stroop were powered to detect BP effects in negative affect at those levels. Only Stroop was sufficiently sensitive to detect differences in stress at 150 participants with the other two tasks requiring a reasonably high numbers of participants. In terms of episodic memory, only spatial memory was sensitive to BP effects of multiple variables with ~ 150 participants and 10–25 assessments. Pairs required 200 or more participants and free recall required 250 or even 300 participants to reach 0.8 power. Both symbols and numbers would require around 150 participants to detect BP effects, and mental rotation performed quite poorly and never reached adequate levels of power even with 300 participants.

Repeating the same analyses for WP effects revealed low power across all variables, which confirms prior accounts that a rather large number of repeated assessments is required to reliably detect these subtle within-person associations which tend to have small effect sizes (Wright et al., 2025). These power curves are included in the supplementary materials.

**Fig. 7:**
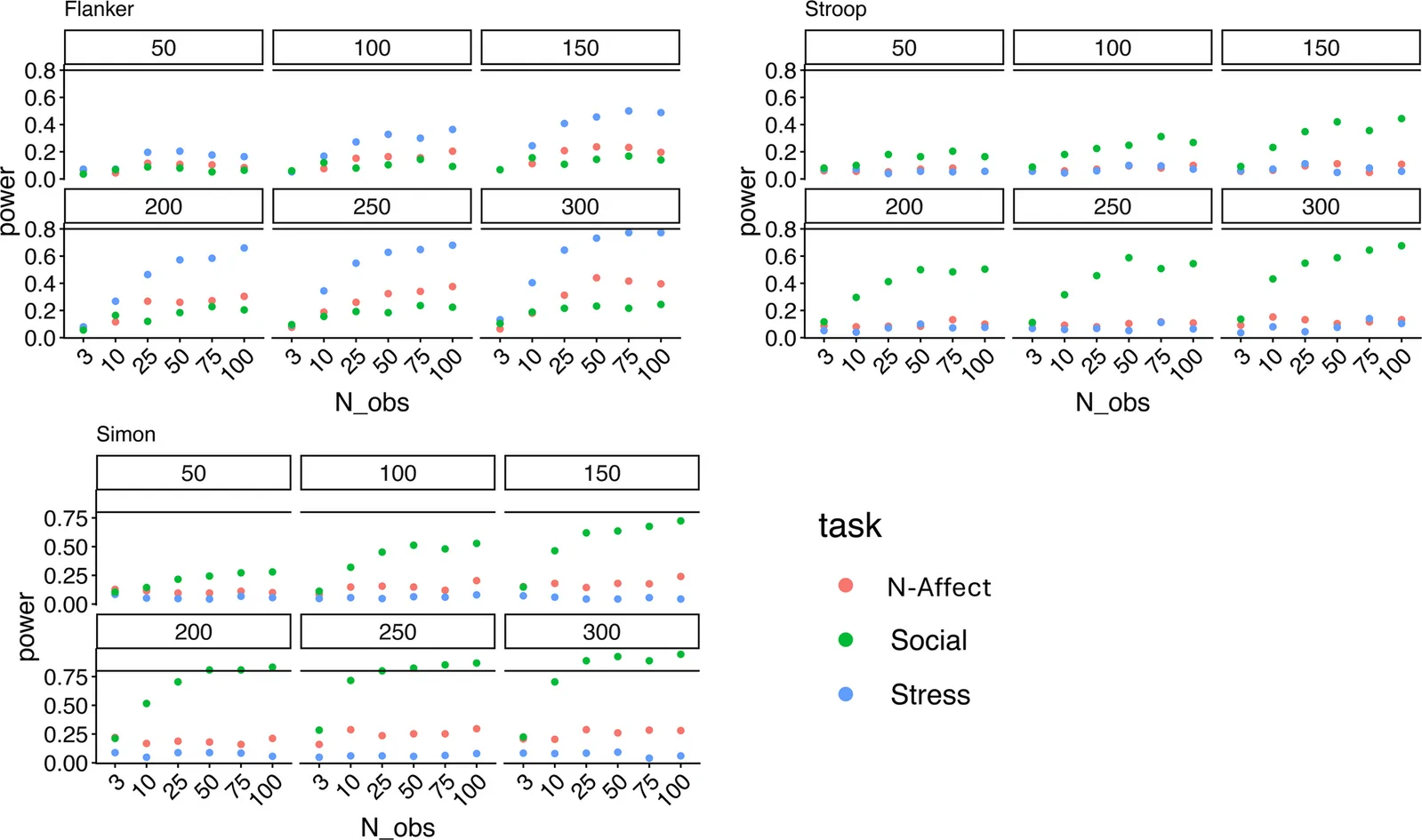
Power curves of the RMSSD for the attentional control tests to detect between-person effects of each contextual variable (negative affect, social interaction, perceived stress)

**Fig. 8:**
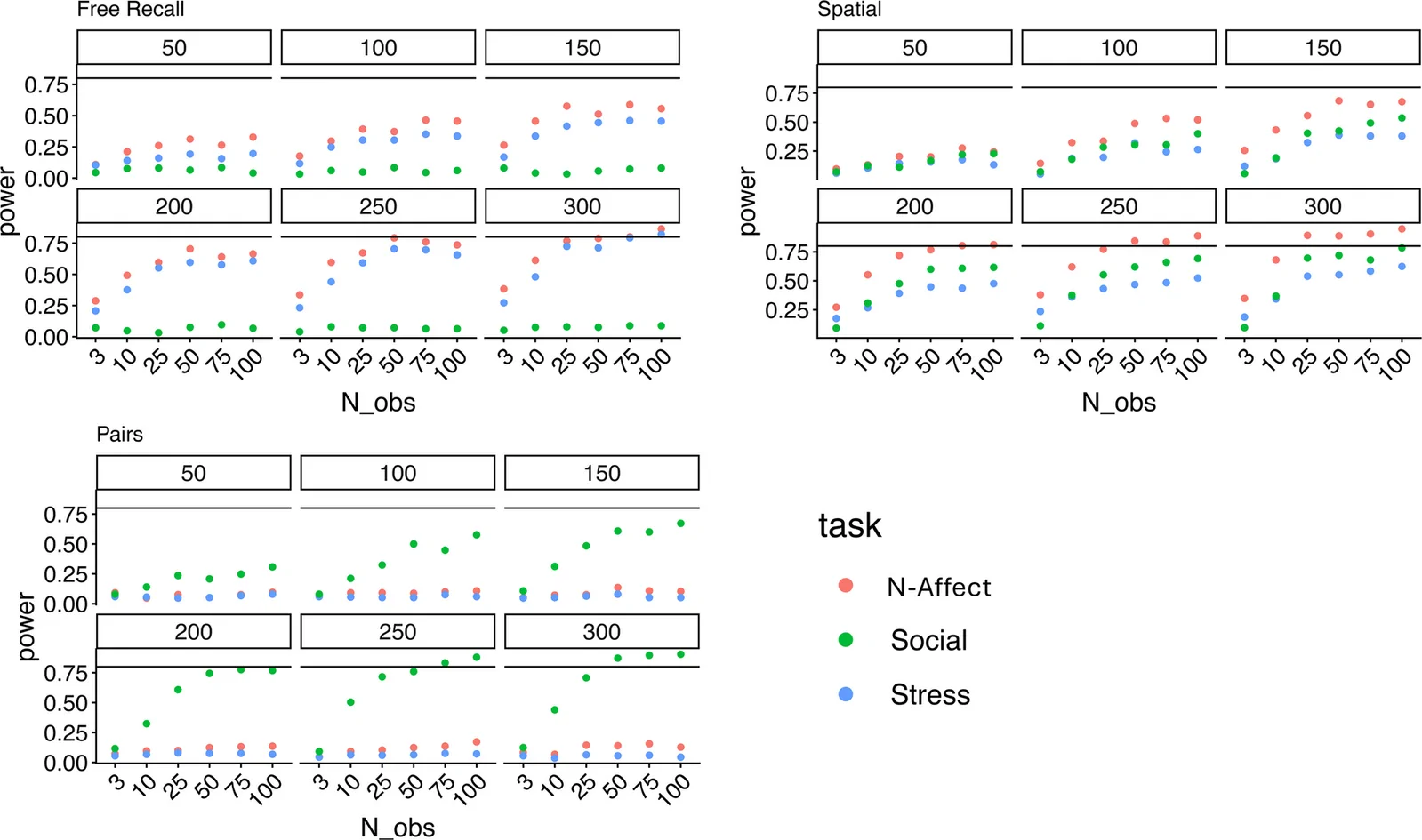
Power curves of the RMSSD for the episodic memory tests to detect between-person effects of each contextual variable (negative affect, social interaction, perceived stress)

**Fig. 9:**
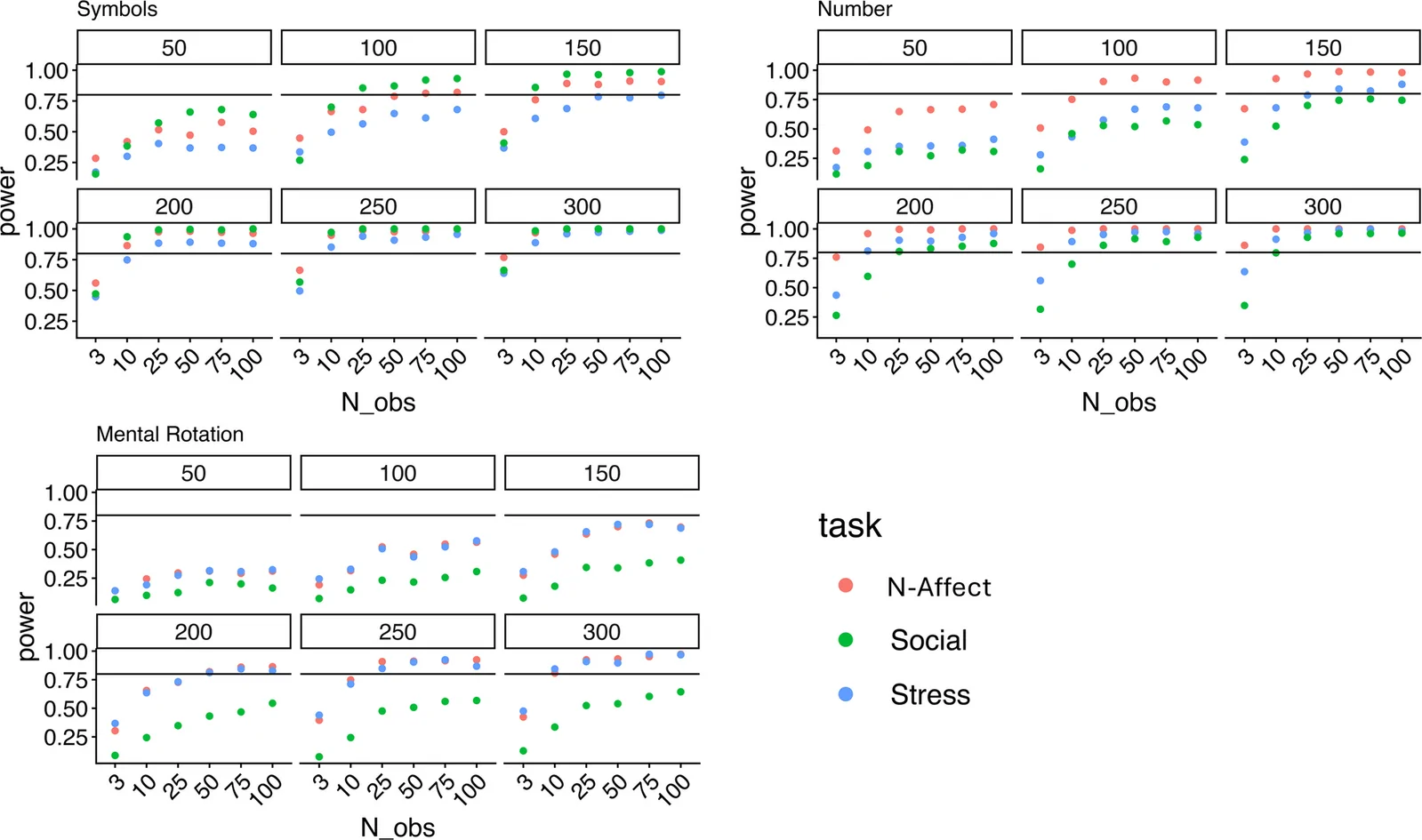
Power curves of the RMSSD of the processing speed tests to detect between-person effects of each contextual variable (negative affect, social interaction, perceived stress)

Similarly, the RMSSD for the attentional control tasks did not reach adequate power under any of our simulation scenarios, with a single exception, that of the Simon task and frequency of social interactions (power curves are plotted in Fig. 7). Two of the memory tests (free recall and spatial) began to approach 0.8 power with around 200 participants and at least 25 repeated assessments (see Fig. 8). Interestingly, both symbols and number comparison began to reach sufficient power with relatively modest sample sizes (e.g., 150 participants and 25 assessments), and even mental rotation achieved good power but at slightly higher sample sizes. This raises the intriguing possibility that variability in processing speed per se (and not other cognitive domains) is uniquely sensitive to contextual variation (see Fig. 9).

## Discussion

High-frequency assessments of cognitive and psychological phenomena are becoming common and afford many practical advantages including increased reliability of mean estimates (Nicosia et al., 2022a; Sliwinski et al., 2018) and the ability to monitor performance in daily life. Numerous studies have shown that when measured repeatedly over short intervals, cognitive performance can vary, at times dramatically (Aschenbrenner & Jackson, 2025). It is assumed that deviations from typical performance reflects a meaningful perturbation of cognitive processing (e.g., due to stress, sleep quality, momentary affect) and is not simply measurement noise. This burgeoning literature could have direct implications for research studies on several levels. The importance of this notion, and therefore the benefits of HFCA, can be illustrated with a few, relatively simple examples.

Behavioral intervention studies that target Alzheimer disease risk factors such as stress, physical activity, and social engagement (Baker et al., 2025; Lenze et al., 2022; Ngandu et al., 2015) are primarily interested in between-person differences (i.e., between those who received an intervention and those who did not). Such studies typically measure baseline cognitive performance and repeat the assessment at regular intervals (e.g., every 6 or 12 months). As we and others have shown, each single assessment is “contaminated” to a certain extent by within-person fluctuations in cognition. Thus, estimates of the intervention effect are noisy, and consequently large sample sizes are needed (Zammit et al., 2025). HFCA can enhance the reliability of mean estimates (as evidenced by the ICCs in our analysis), which directly increases power to detect effects of interest (Wang et al., 2024). As previously discussed, many groups are interested in cognitive variability by examining how much an individual fluctuates from day to day as a marker of neurodegenerative disease risk (e.g., Aschenbrenner et al., 2024; Cerino et al., 2021). One could even address how two constructs covary together over time (Montpetit et al., 2010), for example by examining how much cognition might be harmed in the presence of a stressful event (Sliwinski et al., 2006). Thus, HFCA affords the ability to test many interesting and complex hypotheses, but even relatively simple hypotheses that address only mean-level performance can benefit greatly from an HFCA design.

With a full understanding of these benefits, researchers are faced with decisions regarding which tasks to employ in their study. Presumably, one is interested in measuring underlying cognitive constructs, as opposed to task specific performance, and hence tasks presumed to measure a given domain should correlate within and across people. If one is interested in measuring meaningful fluctuations over time, tasks should evince at least moderate within-person reliability and should covary with changes in the environment (e.g., stress). There is little psychometric evidence available to understand which tasks show these properties. The primary goal of this project was to provide this essential psychometric information by administering a large number of cognitive tests to a sample of adults over a relatively long interval (30 days). While our results clearly have many practical implications for researchers wishing to engage in HFCA testing, there are substantial theoretical contributions as well. We discuss each of these in turn.

First, practically speaking, this study expanded on prior work by providing a comprehensive examination of attentional control. We were interested particularly in the construct of attention not only because of its relationship to aging and Alzheimer disease (Balota et al., 2010) but also the known relationship with attention and aspects of daily life (Aschenbrenner et al., 2022; Draheim et al., 2022; Unsworth, 2015). This expansion of HFCA to the study of attention was quite fortuitous, as our attentional control measures were consistently the best-performing outcomes relative to our other measures, exhibiting high reliability (at both the between- and within-person level), strong inter-task correlations (again at both the between- and within-person level), and consistent relationships with all three of our contextual factors of interest. We argue that attentional control should continue to be emphasized in studies of cognition in daily life.

Tasks from the episodic domain were similarly well-performing. Specifically, all three memory tests exhibited strong between- and within-person reliability as well as between-task correlations (i.e., participants who scored high on free recall also scored high on spatial memory), yet the within-person correlations were notably small (being high on free recall today does not imply you are correspondingly high on spatial memory today). Memory is a rather difficult construct to measure appropriately (Brady et al., 2023) and becomes even more complex upon repeated assessment, where differential strategy use may come into play (Allaire & Marsiske, 2005; Aschenbrenner & Jackson, 2025). For example, it may be easier to employ traditional memory techniques (such as the method of loci) to the pairs test, as these included shopping items, and one could organize a list based on typical progress through a grocery store. Mnemonic techniques may be less straightforward for unrelated word lists, as in our free recall task. We did not explicitly measure strategy use in our tasks, so this is only a speculation, but one that has support in the literature (Ghisletta et al., 2018; Hertzog et al., 2017; Shing et al., 2012).

The results from the processing speed tasks were more mixed. Although all three measures exhibited high between-person reliability and strong between-person correlations, the within-person reliability was extremely low, and the within-person correlations were modest. These issues can be traced to two factors. First, mental rotation had very poor psychometric properties and was a highly skewed variable overall, tending not to correlate strongly with the other processing speed tests, whereas both symbols and number comparison were tightly correlated. Second, all processing speed tasks included 12 trials total per session, similar to other HFCA studies that have used similar tasks (Cerino et al., 2021; Nicosia et al., 2022a). This likely generated a rather noisy estimate at the session level, preventing strong within-person correlations. Moreover, processing speed tests may be unduly influenced by differences across devices (Nicosia et al., 2022b). Future work should consider expanding the number of trials or utilizing alternative outcome metrics than median response time (e.g., a throughput measure similar to our attentional control tasks).

More broadly, these results can be used to select the “optimal” number and type of tests to fit specific research goals. For example, Table 1 suggests that at least 10 assessments are needed to achieve adequate between-person reliability in all tests. As mentioned earlier, the attention tests in particular were sensitive to between-person differences in all three outcomes of interest, and the power figures will guide selection on the number of participants versus number of repeated assessments. These figures also quantify the benefit of repeated testing. For example, the flanker test reached 0.8 power to detect between-person differences in negative affect with only 10 repeated assessments and 100 participants. If only single assessments are given, 200 participants would be required. Arguably, it is more cost-effective to obtain repeated sampling as opposed to doubling the overall sample size. Importantly, power to detect within-person effects was very low for all tasks. This accords with recent work suggesting 100 or more assessments are needed to reliably detect correlations among two covarying factors (Wright et al., 2025).

Theoretically speaking, Table 3 is interesting in showing that all nine tests were relatively equally correlated with one another at the initial assessment. This may suggest some underlying latent factor dominating performance (e.g., general intelligence, familiarity with cognitive testing, familiarity with use of technology). After up to 45 repeated assessments, however, this general factor becomes less prominent, and the tests began to cluster into their respective cognitive domains (yet the attention and memory tests remained rather highly correlated). This was particularly true for the attention tests, but surprisingly, the episodic memory measures remained only modestly correlated. Symbols and number comparison were moderately correlated, as expected. Negative affect, stress, and social interactions showed broadly consistent associations with cognition at the between-person level, although the magnitude of effects did vary across tasks. These patterns suggest that multiple cognitive tests, particularly those with larger and most consistent effects, may be useful in intervention research targeting these lifestyle factors.

### What about variability?

The preceding discussion focused on mean performance, and many researchers are also focused on cognitive variability, defined in our study as the root mean square of successive differences across repeated sessions. First, and as expected, there was substantial daily variation in all tasks, ranging in size from about 20% to almost 40% of the task mean. Again, variability in the attention tasks was strongly correlated at the between-person level (Table 5), with more modest correlations among the processing speed tests. Memory task variability was not strongly correlated, consistent with our prior work (Aschenbrenner & Jackson, 2025), and again could perhaps be a reflection of variation in strategy use across the three tests. Only variability in the processing speed tests were associated with between-person differences in contextual factors, and only those tests were powered to detect between-person differences with a reasonable number of participants and assessments.^1^ We have found in our other work that daily variability in processing speed (and not in other tests) was predictive of Alzheimer disease risk (Aschenbrenner et al., 2024), and others have shown that processing speed variability is critical in separating mild cognitive impairment from healthy aging (Cerino et al., 2021). We had previously assumed this particular task (symbols) also heavily captured attentional control which was producing the relationships with Alzheimer disease. The current results seem to speak against that hypothesis, as attention control variability per se (based on Flanker, Stroop, and Simon tasks) did not correlate with any contextual variable we examined here.

### What task(s) should be used moving forward?

The domains selected for any given study should, obviously, be guided by theory (i.e., I expect this outcome to influence memory due to a specific mechanistic pathway). If theory dictates a specific domain, any task within that domain was largely equal in our study. The sole exception would be the mental rotation task, which we would not recommend as a measure of processing speed (symbols and numbers were far superior). In the absence of theoretical guidance as to the domains, we can strongly recommend any of the three attention tasks we explored here, all of which showed strong reliability and cohesion across multiple levels. While this is likely due in no small part to the extensive development work undertaken by the developers of these tasks (Burgoyne et al., 2023), we also argue that attentional control is simply an essential component of day-to-day functioning, and thus is most likely to pick up on variations within the environment.

### How many assessments and how many people?

Despite the theoretical benefits of HFCAs, they are costly and burdensome to set up and run. Thus, it makes sense to target the minimum number of participants and assessments possible. Of course, firm recommendations that would cover a range of study designs is impossible. Nevertheless, general recommendations depending upon the level of analysis are warranted. For between-person effects (average scores on cognition associated with average scores on contextual variables), 100–200 persons with at least 10 assessments each seems a reasonable starting point. Our power analyses for within-person effects (does a bad contextual effect right now correspond to poor cognition right now) revealed low power across a range of persons by assessment combinations, with at least 300 participants or 100 individual assessments still yielding very low power, consistent with other intensive longitudinal findings in other domains (Wright et al., 2025). While these numbers may seem prohibitively high, it is important to emphasize that the estimates will be influenced by the reliability of the contextual factors themselves (stress, affect, etc.), which we are unable to determine. Moreover, most individuals simply do not experience wide swings in stress or negative affect across a month of observation. These results should not preclude the examination of within-person effects that exhibit more stable fluctuations, for example cortisol (Buchanan et al., 1999). Examinations of cognitive variability will require the largest sample sizes, unless one focuses on processing speed tasks, for which approximately 100 individuals may suffice.

There were many strengths to this study, including the large and diverse sample and large number of cognitive tests examined relative to other studies. Nevertheless, some limitations should be noted. First, participants were required to have an internet-compatible device and commit to three times daily testing for 30 days. Thus, our sample likely excluded a large segment of the population that cannot meet those requirements (e.g., schoolteachers cannot pause in the middle of the day to take a test). We were very flexible in when participants were allowed to take the testing and granted up to 4 h after the initial notification. Thus, we did not sample stress and affect randomly but rather sampled instances when individuals were willing to engage in cognitive testing. Forcing cognitive testing at specific points in time (as with more traditional ecological momentary assessments) may reveal different patterns of effects. Our goal to reach a wide number of people produced a variety of devices utilized to take the testing. Differences in device characteristics could introduce subtle biases in the results. A large body of literature, however, does suggest that key psychological phenomena can be faithfully replicated in online settings (Crump et al., 2013) and are even sufficiently robust as to be useful for computational modeling approaches, including the diffusion model (Ratcliff & Hendrickson, 2021). Moreover, variability introduced by devices should ultimately even out across the minimum of 45 sessions administered here. Similarly, there was a wide age range among participants in our sample. While we consider this a major strength, it should also be noted that psychometric characteristics and other metrics may ultimately change across the lifespan. Variability was quantified in our study as the RMSSD metric, which precludes the calculation of within-person effects. Future work can use more advanced modeling approaches, such as mixed-effects location scale models, to directly assess the influence of *daily* as opposed to average stress on variability. Finally, we did not obtain any measures of digital fluency or smartphone use in our participants. There may be important differences in these characteristics that are subtly biasing our findings.

## Conclusions

This study joins a growing body of literature highlighting the necessity of repeated assessments of cognition in daily life with a particular emphasis on the superiority of utilizing measures of attentional control. Moreover, this study represents one of the largest and longest HFCA studies to date, and the open-source tasks will undoubtedly serve as a valuable resource for investigators to employ HFCA testing in their own work.

## Supplementary Information

Below is the link to the electronic supplementary material.

Supplementary file1 (PDF 428 kb)

## Data Availability

All study data are posted on the Open Science Framework (https://osf.io/tev9z/overview?view_only=5b1ce4ae96a74df1b04c8379b36e0335). The cognitive tasks used in this study have been posted to the Gorilla repository and are accessible at this link (https://app.gorilla.sc/openmaterials/1153479). This study was not preregistered.

## Footnotes

1 One may be concerned that the variability effects in processing speed may be due to device characteristics (e.g., differences in processor power), which would manifest more strongly on measures of response time than accuracy. Thus, to ensure findings listed in Table 7 were not spurious due to differences in devices, we conducted a sensitivity analysis where we examined only smartphone responses. Effect size estimates were similar across the full sample (Table 7) and the smartphone-only subsample (included in the supplement).

## Code availability

All analysis code is available on the Open Science Framework (https://osf.io/tev9z/overview?view_only=5b1ce4ae96a74df1b04c8379b36e0335).
