# Supplementary material for: Developing a psychological test battery to measure cognition in daily life
Source: Behav Res Methods. 2026 Jul 24;58(9):247. doi: 10.3758/s13428-026-03128-4 (PMC13400683; doi:10.3758/s13428-026-03128-4)
Supplement: Supplementary file 1 — Supplementary file1 (PDF 428 kb) [file 13428_2026_3128_MOESM1_ESM.pdf]

Further Details on the tasks used in the CVB.

Stroop Squared Task: At the start of each implementation of this task, the participant first viewed an instruction screen with a single example to remind them of the task procedures. After clicking a “Continue” button, they were presented with a “Get Ready” screen for 5000 milliseconds to allow them to prepare for the main task. On each trial, the following events occurred: a fixation cross was presented in the center of the screen for 100 ms, a blank screen for 100 ms, then the target and response options were displayed as described in the main text. Once a response was chosen, accuracy feedback (green check or red x) was displayed for 200 ms followed by 100 ms blank screen prior to the start of the next trial. There was no trial level time outs, that is, trials did not advance until a response was made. The task ran for exactly 90 seconds. The task design generated four “conditions” based on crossing two factors, congruency of the target (is the target word and the color ink the same or different) and congruency of the responses (are the response words and color ink the same or different). Item types were sampled randomly with replacement, thus over the course of the task each condition will be represented equally often but there may be differential repeats of trial types across individuals.

Flanker Squared Task: At the start of each implementation of this task, the participant first viewed an instruction screen with a single example to remind them of the task procedures. After clicking a “Continue” button, they were presented with a “Get Ready” screen for 5000 milliseconds to allow them to prepare for the main task. On each trial, the following events occurred: a fixation cross was presented in the center of the screen for 100 ms, a blank screen for 100 ms, then the target and response options were displayed as described in the main text. Once a response was chosen, accuracy feedback (green check or red x) was displayed for 200 ms followed by 100 ms blank screen prior to the start of the next trial. There was no trial level time outs, that is, trials did not advance until a response was made. The task ran for exactly 90 seconds. The task design generated four “conditions” based on crossing two factors, congruency of the target (the central and flanking arrows of the target point in the same or different directions) and congruency of the responses (the central and flanking arrows of the target point in the same or different directions). Item types were sampled randomly with replacement, thus over the course of the task each condition will be represented equally often but there may be differential repeats of trial types across individuals.

Simon Squared Task: At the start of each implementation of this task, the participant first viewed an instruction screen with a single example to remind them of the task procedures. After clicking a “Continue” button, they were presented with a “Get Ready” screen for 5000 milliseconds to allow them to prepare for the main task. On each trial, the following events occurred: a fixation cross was presented in the center of the screen for 100 ms, a blank screen for 100 ms, then the target and response options were

For Review Only

displayed as described in the main text. Once a response was chosen, accuracy feedback (green check or red x) was displayed for 200 ms followed by 100 ms blank screen prior to the start of the next trial. There was no trial level time outs, that is, trials did not advance until a response was made. The task ran for exactly 90 seconds. The task design generated four “conditions” based on crossing two factors, congruency of the target (is the target arrow direction and screen location the same or different) and congruency of the responses (is the response word and response location the same or different). Item types were sampled randomly with replacement, thus over the course of the task each condition will be represented equally often but there may be differential repeats of trial types across individuals.

Free Recall Task: After a single instruction screen, 5 words were displayed on screen for exactly 15 seconds after which the next set of 5 was shown with no delay. Once all 15 items were displayed, a free recall box was displayed and participants had unlimited time to record as many items as they could remember. A self-paced interim screen (“You will now see the words again”) was presented prior to the second round of items which occurred in the same order as before.

Paired Associates Task: After a single instruction screen, 5 items and prices were displayed on screen for exactly 15 seconds after which the next set of 5 was shown with no delay. Once all 10 items were displayed, they immediately entered the recall phase. During this phase the following events occurred: a fixation cross was shown for 750 ms followed by a randomly generated item from the study set and a 9 digit keypad. Participants had unlimited time to input a response which generated a feedback response (green check mark for correct response) for 800 ms. After all 10 items were presented in the test phase, a self-paced interim screen was shown followed by a second round of study. During the second study phase the items were presented in the same order as the first study phase but the order of items at test were different due to randomization.

Spatial Memory Task: After a single instruction screen, a “Get Ready” screen was displayed for 5000ms. Then all 6 study items were displayed in the grid for 8 seconds followed by a “you will now start the test” screen for 5000 ms. Items were randomly presented one at a time for participants to place back in the grid. After placing an item accuracy feedback was displayed for 1000 ms followed immediately by the next test trial. During the second study phase, items were presented in the same locations but the order of items at test was again randomized.

Symbols Task: At the start of each implementation of this task, the participant first viewed an instruction screen with a single example to remind them of the task procedures. After clicking a “Continue” button, they were presented with a “Get Ready” screen for 5000 milliseconds to allow them to prepare for the main task. On each trial,

For Review Only

the following events occurred, a blank screen ITI for 1000 ms, followed by a task trial. Each trial was displayed until a response was made. Accuracy feedback was displayed for 200 ms after a response was made. 12 trials were presented in total. Item sets and response options were generated randomly on each trial.

Number Comparison Task: At the start of each implementation of this task, the participant first viewed an instruction screen with a single example to remind them of the task procedures. After clicking a “Continue” button, they were presented with a “Get Ready” screen for 2000 milliseconds to allow them to prepare for the main task. On each trial, the following events occurred, a fixation cross for 500 ms, followed by a task trial. Each trial was displayed until a response was made. Accuracy feedback was displayed for 200 ms after a response was made. 12 trials were presented in total. Item sets and response options were generated randomly on each trial. Within each administration of the task, equal numbers of “correct” and “incorrect” items were displayed. The mismatching digit was always off by “one” and the location (first digit, second digit) was equally represented across trials. Within each administration the items were selected randomly.

Mental Rotation Task: At the start of each implementation of this task, the participant first viewed an instruction screen with a single example to remind them of the task procedures. After clicking a “Continue” button, they were presented with a “Get Ready” screen for 2000 milliseconds to allow them to prepare for the main task. On each trial, the following events occurred, a fixation cross for 500 ms, followed by a task trial. Each trial was displayed until a response was made. Accuracy feedback was displayed for 200 ms after a response was made. 12 trials were presented in total. Item sets and response options were generated randomly on each trial. Within each administration of the task, equal numbers of “correct” and “incorrect” items were displayed. Within each administration the items were selected randomly.

For Review Only

Full output for all models in Analysis 2

Between and within-person effects on mean scores for each task

Flanker

	**flanker score**			**flanker score**			**flanker score**		
Predictors	*Estimates*	*CI*	*p*	*Estimates*	*CI*	*p*	*Estimates*	*CI*	*p*
(Intercept)	55.25	41.95 – 68.55	**<0.001**	63.81	51.65 – 75.97	**<0.001**	32.46	24.27 – 40.64	**<0.001**
stress wp	-0.51	-1.03 – 0.01	0.054						
stress bp	-4.04	-7.63 – -0.45	**0.028**						
age	-0.31	-0.49 – -0.12	**0.001**	-0.37	-0.54 – -0.20	**<0.001**	-0.24	-0.39 – -0.09	**0.002**
neg wp				-0.55	-1.11 – 0.01	0.056			
neg bp				-8.21	-12.25 – -4.18	**<0.001**			
social wp							-0.55	-1.28 – 0.17	0.134
social bp							20.03	9.16 – 30.89	**<0.001**
**Random Effects**									
σ^2	114.42			114.34			115.70		
τ_{00}	283.32 $_{id}$			270.03 $_{id}$			271.45 $_{id}$		
τ_{11}	4.47 $_{id.stress\_wp}$			4.70 $_{id.neg\_wp}$			6.32 $_{id.social\_wp}$		
ρ_{01}	-0.06 $_{id}$			0.25 $_{id}$			-0.45 $_{id}$		
ICC	0.71			0.70			0.70		
N	139 $_{id}$			139 $_{id}$			139 $_{id}$		

For Review Only

Observations	7521	7521	7521
Marginal R^2 / Conditional R^2	0.055 / 0.730	0.104 / 0.735	0.089 / 0.729

Stroop

	stroop score			stroop score			stroop score		
Predictors	*Estimates*	*CI*	*p*	*Estimates*	*CI*	*p*	*Estimates*	*CI*	*p*
(Intercept)	58.25	46.20 – 70.30	**<0.001**	63.81	52.69 – 74.94	**<0.001**	36.03	28.44 – 43.62	**<0.001**
stress wp	-0.26	-0.75 – 0.23	0.293						
stress bp	-4.51	-7.77 – -1.26	**0.007**						
age	-0.25	-0.41 – -0.08	**0.004**	-0.27	-0.43 – -0.12	**0.001**	-0.18	-0.32 – -0.03	**0.015**
neg wp				-0.58	-1.12 – -0.03	**0.038**			
neg bp				-7.87	-11.56 – -4.18	**<0.001**			
social wp							-0.63	-1.28 – 0.02	0.058
social bp							16.64	6.57 – 26.70	**0.001**
**Random Effects**									
σ^2	108.71			108.91			110.07		
τ_{00}	233.31 $_{id}$			222.21 $_{id}$			227.22 $_{id}$		
τ_{11}	3.77 $_{id.stress\_wp}$			4.28 $_{id.neg\_wp}$			3.78 $_{id.social\_wp}$		
ρ_{01}	-0.11 $_{id}$			0.16 $_{id}$			-0.37 $_{id}$		

For Review Only

ICC	0.68			0.67			0.67		
N	139 $_{id}$			139 $_{id}$			139 $_{id}$		
Observations	7581			7581			7581		
Marginal R^2 / Conditional R^2	0.049 / 0.700			0.089 / 0.702			0.065 / 0.696		

Simon

	simon score			simon score			simon score		
Predictors	*Estimates*	*CI*	*p*	*Estimates*	*CI*	*p*	*Estimates*	*CI*	*p*
(Intercept)	62.19	50.34 – 74.05	**<0.001**	65.25	54.17 – 76.33	**<0.001**	41.62	34.46 – 48.78	**<0.001**
stress wp	-0.04	-0.54 – 0.46	0.867						
stress bp	-3.53	-6.73 – -0.33	**0.031**						
age	-0.08	-0.24 – 0.09	0.352	-0.09	-0.25 – 0.06	0.234	-0.04	-0.17 – 0.09	0.581
neg wp				-0.86	-1.47 – -0.25	**0.006**			
neg bp				-5.54	-9.22 – -1.87	**0.003**			
social wp							0.04	-0.75 – 0.82	0.922
social bp							19.70	10.20 – 29.20	**<0.001**
**Random Effects**									
σ^2	130.02			129.35			130.57		

For Review Only

τ_{00}	228.50 $_{id}$	222.14 $_{id}$	209.20 $_{id}$
τ_{11}	3.39 $_{id.stress\_wp}$	5.45 $_{id.neg\_wp}$	8.11 $_{id.social\_wp}$
ρ_{01}	-0.23 $_{id}$	0.22 $_{id}$	-0.48 $_{id}$
ICC	0.64	0.63	0.62
N	139 $_{id}$	139 $_{id}$	139 $_{id}$
Observations	7534	7534	7534
Marginal R^2 / Conditional R^2	0.020 / 0.647	0.037 / 0.648	0.065 / 0.642

For Review Only

Free Recall

	**fr correct**			**fr correct**			**fr correct**		
Predictors	*Estimates*	*CI*	*p*	*Estimates*	*CI*	*p*	*Estimates*	*CI*	*p*
(Intercept)	19.74	14.92 – 24.56	**<0.001**	20.60	16.11 – 25.10	**<0.001**	14.35	11.28 – 17.42	**<0.001**
stress wp	-0.20	-0.37 – -0.04	**0.017**						
stress bp	-1.32	-2.62 – -0.02	**0.047**						
age	-0.09	-0.16 – -0.02	**0.007**	-0.10	-0.16 – -0.03	**0.003**	-0.06	-0.12 – -0.01	**0.027**
neg wp				-0.40	-0.59 – -0.20	**<0.001**			
neg bp				-1.94	-3.44 – -0.45	**0.011**			
social wp							-0.26	-0.55 – 0.03	0.081
social bp							2.52	-1.55 – 6.58	0.225

Random Effects

σ^2	16.69	16.59	16.67
τ_{00}	37.12 $_{id}$	36.35 $_{id}$	37.50 $_{id}$
τ_{11}	0.32 $_{id.stress\_wp}$	0.48 $_{id.neg\_wp}$	1.14 $_{id.social\_wp}$
ρ_{01}	0.04 $_{id}$	0.21 $_{id}$	-0.36 $_{id}$
ICC	0.69	0.69	0.69
N	139 $_{id}$	139 $_{id}$	139 $_{id}$
Observations	7298	7298	7298
Marginal R^2 / Conditional R^2	0.037 / 0.702	0.050 / 0.703	0.027 / 0.702

For Review Only

Spatial

	**spatial correct**			**spatial correct**			**spatial correct**		
Predictors	*Estimates*	*CI*	*p*	*Estimates*	*CI*	*p*	*Estimates*	*CI*	*p*
(Intercept)	11.55	9.84 – 13.27	**<0.001**	12.57	11.02 – 14.13	**<0.001**	8.54	7.47 – 9.60	**<0.001**
stress wp	-0.02	-0.12 – 0.09	0.760						
stress bp	-0.56	-1.02 – -0.09	**0.018**						
age	-0.05	-0.08 – -0.03	**<0.001**	-0.06	-0.08 – -0.04	**<0.001**	-0.04	-0.06 – -0.02	**<0.001**
neg wp				-0.10	-0.22 – 0.01	0.070			
neg bp				-1.09	-1.60 – -0.57	**<0.001**			
social wp							-0.27	-0.46 – -0.08	**0.006**

social bp							2.32	0.91 – 3.74	**0.001**
**Random Effects**									
σ^2	7.00			7.01			6.95		
τ_{00}	4.62 $_{id}$			4.29 $_{id}$			4.41 $_{id}$		
τ_{11}	0.11 $_{id.stress\_wp}$			0.10 $_{id.neg\_wp}$			0.54 $_{id.social\_wp}$		
ρ_{01}	-0.07 $_{id}$			0.27 $_{id}$			-0.32 $_{id}$		
ICC	0.40			0.38			0.39		
N	139 $_{id}$			139 $_{id}$			139 $_{id}$		
Observations	7528			7528			7528		
Marginal R^2 / Conditional R^2	0.053 / 0.433			0.082 / 0.433			0.062 / 0.431		

Pairs

	**pairs correct**			**pairs correct**			**pairs correct**		
Predictors	*Estimates*	*CI*	*p*	*Estimates*	*CI*	*p*	*Estimates*	*CI*	*p*
(Intercept)	14.60	11.49 – 17.72	**<0.001**	16.19	13.31 – 19.07	**<0.001**	11.53	9.59 – 13.47	**<0.001**
stress wp	-0.03	-0.17 – 0.11	0.710						
stress bp	-0.54	-1.39 – 0.30	0.204						
age	-0.04	-0.09 – -0.00	**0.042**	-0.06	-0.10 – -0.02	**0.005**	-0.04	-0.07 – -0.00	**0.043**
neg wp				-0.21	-0.36 – -0.06	**0.005**			
neg bp				-1.24	-2.19 – -0.28	**0.011**			

For Review Only

social wp							-0.24	-0.43 – -0.04	**0.016**
social bp							2.76	0.19 – 5.34	**0.036**
**Random Effects**									
σ^2	11.45			11.46			11.53		
τ_{00}	15.46 $_{id}$			14.90 $_{id}$			15.04 $_{id}$		
τ_{11}	0.23 $_{id.stress\_wp}$			0.19 $_{id.neg\_wp}$			0.16 $_{id.social\_wp}$		
ρ_{01}	-0.09 $_{id}$			0.27 $_{id}$			-0.71 $_{id}$		
ICC	0.58			0.57			0.57		
N	139 $_{id}$			139 $_{id}$			139 $_{id}$		
Observations	7547			7547			7547		
Marginal R^2 / Conditional R^2	0.018 / 0.584			0.040 / 0.584			0.029 / 0.579		

Symbols

	symbols rt			symbols rt			symbols rt		
Predictors	*Estimates*	*CI*	*p*	*Estimates*	*CI*	*p*	*Estimates*	*CI*	*p*
(Intercept)	402.20	43.19 – 761.22	**0.028**	326.03	-8.00 – 660.06	0.056	1034.81	797.10 – 1272.53	**<0.001**
stress wp	7.26	-13.47 – 27.98	0.492						
stress bp	154.72	57.73 – 251.72	**0.002**						
age	24.20	19.22 – 29.18	**<0.001**	24.33	19.64 – 29.02	**<0.001**	20.47	16.03 – 24.92	**<0.001**

For Review Only

neg wp				-13.66	-40.62 – 13.30	0.321			
neg bp				223.47	112.64 – 334.31	**<0.001**			
social wp							37.85	6.75 – 68.95	**0.017**
social bp							-261.79	-576.90 – 53.32	0.103
**Random Effects**									
σ^2	218989.16			217143.75			220082.09		
τ_{00}	204012.04 $_{id}$			196421.90 $_{id}$			215714.65 $_{id}$		
τ_{11}	5936.90 $_{id.stress\_wp}$			11892.17 $_{id.neg\_wp}$			10686.80 $_{id.social\_wp}$		
ρ_{01}	-0.07 $_{id}$			-0.06 $_{id}$			-0.04 $_{id}$		
ICC	0.49			0.48			0.50		
N	139 $_{id}$			139 $_{id}$			139 $_{id}$		
Observations	7542			7542			7542		
Marginal R^2 / Conditional R^2	0.256 / 0.618			0.269 / 0.621			0.232 / 0.614		

For Review Only

Numbers

	**number rt**			**number rt**			**number rt**		
Predictors	*Estimates*	*CI*	*p*	*Estimates*	*CI*	*p*	*Estimates*	*CI*	*p*
(Intercept)	231.45	-75.65 – 538.54	0.140	146.65	-137.91 – 431.22	0.312	780.76	576.63 – 984.90	**<0.001**
stress wp	0.43	-13.09 – 13.94	0.951						

stress bp	140.38	57.38 – 223.39	**0.001**						
age	23.47	19.21 – 27.73	**<0.001**	23.58	19.59 – 27.57	**<0.001**	20.01	16.20 – 23.81	**<0.001**
neg wp				-12.88	-28.35 – 2.60	0.103			
neg bp				211.39	116.94 – 305.84	**<0.001**			
social wp							43.68	18.70 – 68.65	**0.001**
social bp							-187.74	-458.48 – 82.99	0.174
**Random Effects**									
σ^2	138629.49			138356.31			137803.89		
τ_{00}	152780.39 $_{id}$			145138.29 $_{id}$			161384.22 $_{id}$		
τ_{11}	1372.57 $_{id.stress\_wp}$			1880.49 $_{id.neg\_wp}$			7352.41 $_{id.social\_wp}$		
ρ_{01}	0.33 $_{id}$			0.29 $_{id}$			0.23 $_{id}$		
ICC	0.53			0.51			0.54		
N	139 $_{id}$			139 $_{id}$			139 $_{id}$		
Observations	7490			7490			7490		
Marginal R^2 / Conditional R^2	0.323 / 0.679			0.338 / 0.678			0.297 / 0.677		

For Review Only

Mental Rotation

	**mental rt**			**mental rt**			**mental rt**		
Predictors	*Estimates*	*CI*	*p*	*Estimates*	*CI*	*p*	*Estimates*	*CI*	*p*

(Intercept)	583.29	-15.00 – 1181.57	0.056	974.10	424.93 – 1523.27	**0.001**	803.12	414.80 – 1191.45	**<0.001**
stress wp	17.99	-14.23 – 50.21	0.274						
stress bp	58.29	-103.37 – 219.95	0.480						
age	29.49	21.20 – 37.78	**<0.001**	26.38	18.67 – 34.08	**<0.001**	27.74	20.48 – 35.00	**<0.001**
neg wp				-56.40	-96.94 – -15.85	**0.006**			
neg bp				-77.67	-260.00 – 104.67	0.404			
social wp							70.64	24.28 – 117.00	**0.003**
social bp							-43.60	-558.45 – 471.24	0.868
**Random Effects**									
σ^2	535291.91			531487.30			538691.58		
τ_{00}	573498.61 $_{id}$			581624.17 $_{id}$			577888.87 $_{id}$		
τ_{11}	14754.98 $_{id.stress\_wp}$			27211.14 $_{id.neg\_wp}$			21209.86 $_{id.social\_wp}$		
ρ_{01}	-0.17 $_{id}$			-0.45 $_{id}$			-0.11 $_{id}$		
ICC	0.52			0.53			0.52		
N	139 $_{id}$			139 $_{id}$			139 $_{id}$		
Observations	7600			7600			7600		
Marginal R^2 / Conditional R^2	0.185 / 0.609			0.182 / 0.614			0.180 / 0.606		

For Review Only

Between-person effects on RMSSD for each task

Flanker

	rmssd			rmssd			rmssd		
Predictors	*Estimates*	*CI*	*p*	*Estimates*	*CI*	*p*	*Estimates*	*CI*	*p*
(Intercept)	18.10	14.39 – 21.81	**<0.001**	16.76	13.22 – 20.30	**<0.001**	16.48	14.08 – 18.88	**<0.001**
stress bp	-0.64	-1.64 – 0.37	0.211						
age	-0.15	-0.20 – -0.10	**<0.001**	-0.14	-0.19 – -0.09	**<0.001**	-0.13	-0.18 – -0.09	**<0.001**
neg bp				-0.28	-1.45 – 0.90	0.643			
social bp							-0.90	-4.08 – 2.29	0.579
Observations	139			139			139		
R^2 / R^2 adjusted	0.220 / 0.208			0.212 / 0.200			0.212 / 0.201		

For Review Only

Stroop

	rmssd			rmssd			rmssd		
Predictors	*Estimates*	*CI*	*p*	*Estimates*	*CI*	*p*	*Estimates*	*CI*	*p*
(Intercept)	15.43	11.95 – 18.91	**<0.001**	14.08	10.80 – 17.36	**<0.001**	17.12	14.89 – 19.34	**<0.001**

stress bp	0.20	-0.74 – 1.14	0.671						
age	-0.12	-0.17 – -0.08	**<0.001**	-0.11	-0.16 – -0.07	**<0.001**	-0.12	-0.17 – -0.08	**<0.001**
neg bp				0.78	-0.31 – 1.86	0.161			
social bp							-2.15	-5.10 – 0.81	0.153
Observations	139			139			139		
R^2 / R^2 adjusted	0.221 / 0.209			0.231 / 0.220			0.231 / 0.220		

Simon

	**rmssd**			**rmssd**			**rmssd**		
Predictors	*Estimates*	*CI*	*p*	*Estimates*	*CI*	*p*	*Estimates*	*CI*	*p*
(Intercept)	17.36	12.82 – 21.90	**<0.001**	16.16	11.88 – 20.45	**<0.001**	20.43	17.54 – 23.33	**<0.001**
stress bp	0.43	-0.80 – 1.66	0.491						
age	-0.16	-0.23 – -0.10	**<0.001**	-0.16	-0.22 – -0.10	**<0.001**	-0.17	-0.22 – -0.11	**<0.001**
neg bp				1.00	-0.42 – 2.42	0.166			
social bp							-3.50	-7.34 – 0.34	0.073
Observations	139			139			139		

For Review Only

R² / R² adjusted	0.232 / 0.221			0.240 / 0.229			0.248 / 0.237		

Free Recall

	rmssd			rmssd			rmssd		
Predictors	*Estimates*	*CI*	*p*	*Estimates*	*CI*	*p*	*Estimates*	*CI*	*p*
(Intercept)	6.75	5.39 – 8.11	**<0.001**	6.91	5.62 – 8.20	**<0.001**	5.73	4.84 – 6.61	**<0.001**
stress bp	-0.26	-0.63 – 0.11	0.164						
age	-0.03	-0.05 – -0.01	**0.001**	-0.03	-0.05 – -0.02	**<0.001**	-0.03	-0.04 – -0.01	**0.002**
neg bp				-0.39	-0.81 – 0.04	0.075			
social bp							0.39	-0.78 – 1.56	0.515
Observations	139			139			139		
R^2 / R^2 adjusted	0.082 / 0.069			0.090 / 0.077			0.072 / 0.058		

Spatial

	rmssd			rmssd			rmssd		
Predictors	*Estimates*	*CI*	*p*	*Estimates*	*CI*	*p*	*Estimates*	*CI*	*p*
(Intercept)	2.70	1.83 – 3.56	**<0.001**	2.52	1.71 – 3.33	**<0.001**	3.78	3.22 – 4.33	**<0.001**
stress bp	0.23	-0.01 – 0.46	0.055						

For Review Only

age	0.01	-0.01 – 0.02	0.409	0.01	-0.01 – 0.02	0.323	0.00	-0.01 – 0.01	0.914
neg bp				0.35	0.08 – 0.62	**0.010**			
social bp							-0.71	-1.45 – 0.03	0.059
Observations	139			139			139		
R^2 / R^2 adjusted	0.027 / 0.013			0.048 / 0.034			0.026 / 0.012		

Pairs

	rmssd			rmssd			rmssd		
Predictors	*Estimates*	*CI*	*p*	*Estimates*	*CI*	*p*	*Estimates*	*CI*	*p*
(Intercept)	4.40	3.57 – 5.22	**<0.001**	4.35	3.57 – 5.13	**<0.001**	4.95	4.43 – 5.47	**<0.001**
stress bp	0.07	-0.16 – 0.29	0.562						
age	-0.01	-0.02 – 0.00	0.214	-0.01	-0.02 – 0.00	0.207	-0.01	-0.02 – 0.00	0.138
neg bp				0.10	-0.16 – 0.36	0.442			
social bp							-0.70	-1.40 – -0.01	**0.047**
Observations	139			139			139		
R^2 / R^2 adjusted	0.026 / 0.011			0.028 / 0.013			0.051 / 0.037		

For Review Only

Symbols

	rmssd			rmssd			rmssd		
Predictors	*Estimates*	*CI*	*p*	*Estimates*	*CI*	*p*	*Estimates*	*CI*	*p*
(Intercept)	202.25	-19.83 – 424.34	0.074	105.47	-98.03 – 308.98	0.307	657.86	518.56 – 797.15	**<0.001**
stress bp	86.03	26.05 – 146.01	**0.005**						
age	2.92	-0.16 – 6.00	0.063	3.40	0.55 – 6.26	**0.020**	1.46	-1.15 – 4.07	0.269
neg bp				145.23	77.74 – 212.73	**<0.001**			
social bp							-369.15	-553.88 – -184.41	**<0.001**
Observations	139			139			139		
R^2 / R^2 adjusted	0.057 / 0.044			0.119 / 0.106			0.104 / 0.091		

For Review Only

Numbers

	rmssd			rmssd			rmssd		
Predictors	*Estimates*	*CI*	*p*	*Estimates*	*CI*	*p*	*Estimates*	*CI*	*p*
(Intercept)	67.13	-149.36 – 283.62	0.541	-26.61	-223.59 – 170.37	0.790	491.58	348.94 – 634.22	**<0.001**
stress bp	100.55	42.09 – 159.02	**0.001**						
age	3.08	0.07 – 6.08	**0.045**	3.50	0.74 – 6.27	**0.013**	0.88	-1.79 – 3.55	0.514

neg bp				162.22	96.89 – 227.55	**<0.001**			
social bp							-208.04	-397.22 – -18.87	**0.031**
Observations	139			139			139		
R^2 / R^2 adjusted	0.079 / 0.066			0.151 / 0.139			0.034 / 0.020		

Mental Rotation

	rmssd			rmssd			rmssd		
Predictors	*Estimates*	*CI*	*p*	*Estimates*	*CI*	*p*	*Estimates*	*CI*	*p*
(Intercept)	132.40	-199.54 – 464.34	0.432	92.43	-219.35 – 404.21	0.559	601.22	385.85 – 816.59	**<0.001**
stress bp	106.70	17.06 – 196.34	**0.020**						
age	8.72	4.11 – 13.32	**<0.001**	8.73	4.35 – 13.11	**<0.001**	6.47	2.44 – 10.51	**0.002**
neg bp				149.02	45.62 – 252.43	**0.005**			
social bp							-258.85	-544.48 – 26.78	0.075
Observations	139			139			139		
R^2 / R^2 adjusted	0.095 / 0.082			0.111 / 0.098			0.080 / 0.066		

For Review Only

Power Curves for the within-person effects in Analysis 3

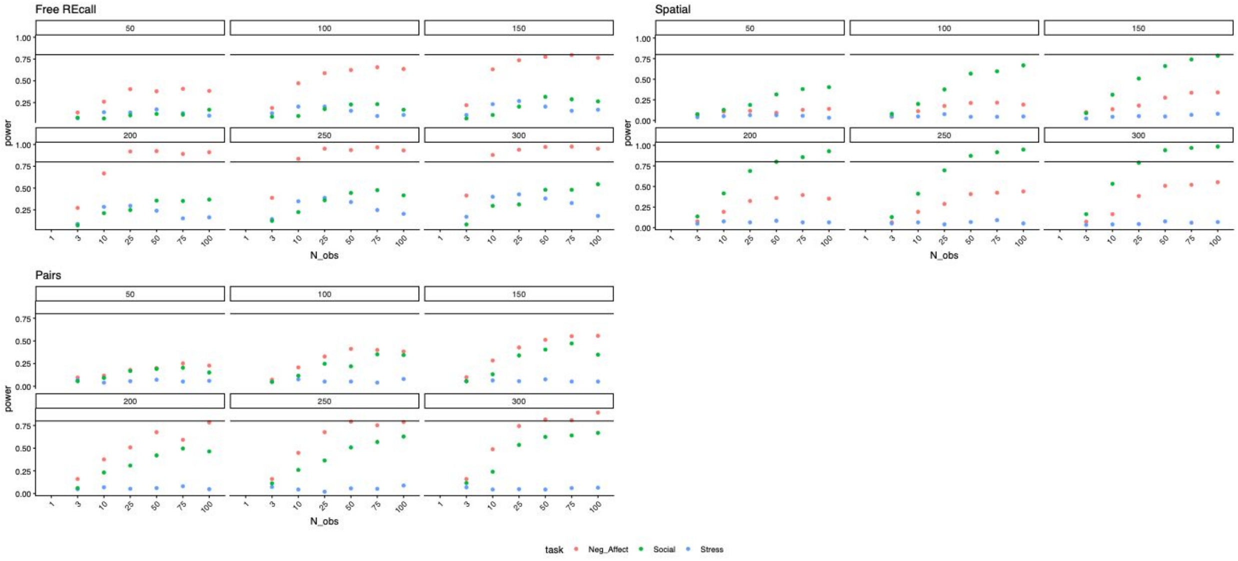

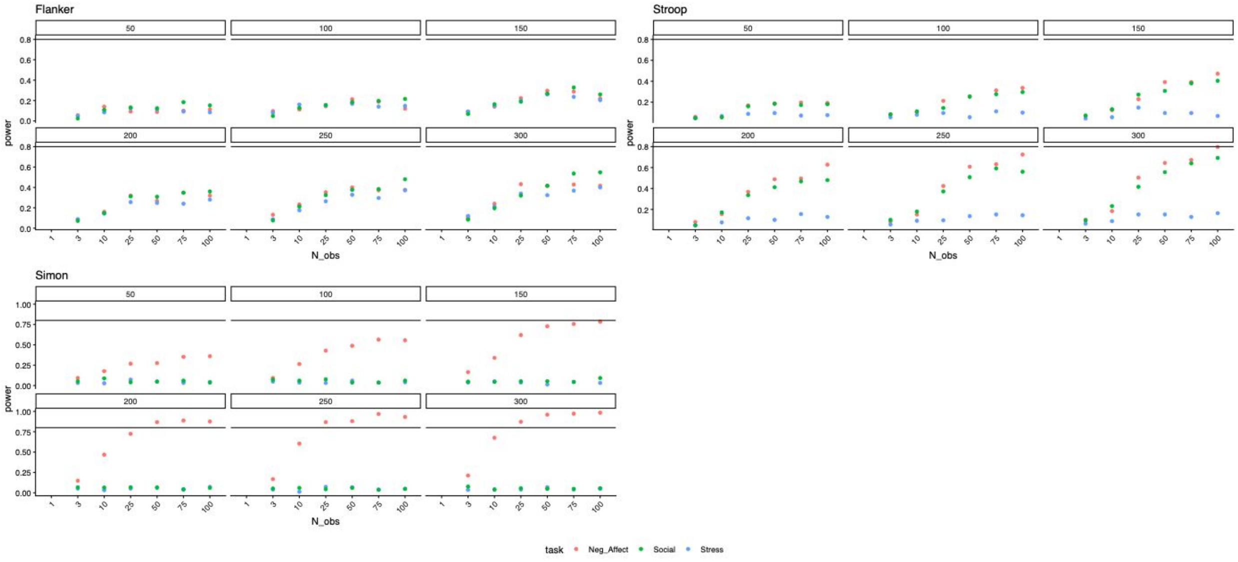

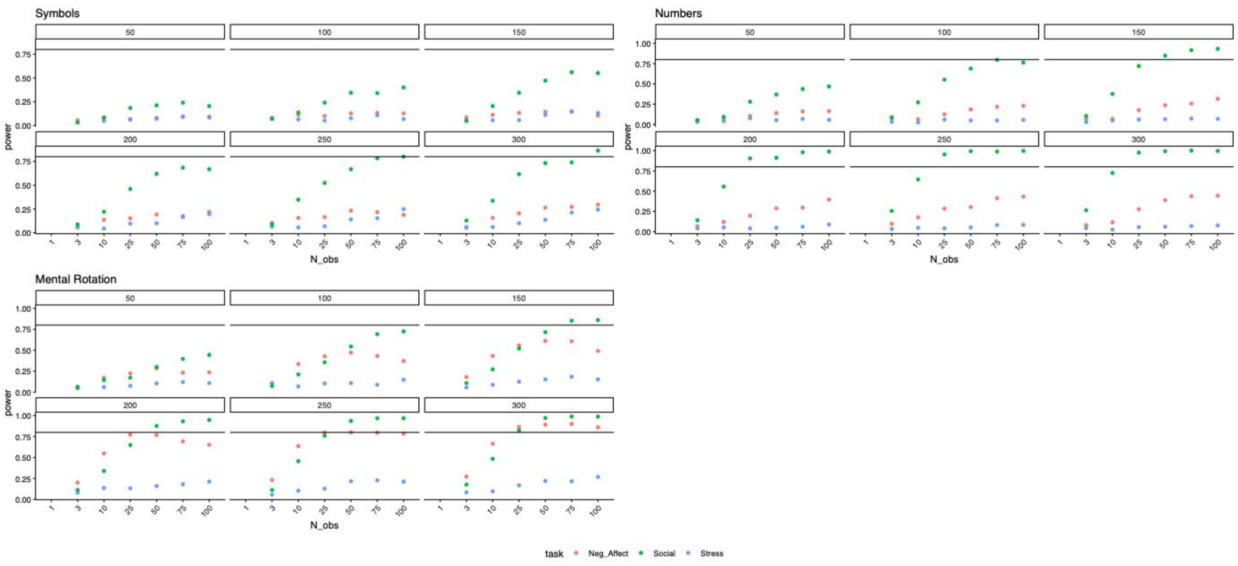
Symbols
Numbers
Mental Rotation
50
100
150
200
250
300
power
N_obs
task
Neg_Affect
Social
Stress

Sensitivity analyses of the processing speed rmssd.

To ensure the findings regarding rmssd in the processing speed tasks are not due to device characteristics, we subset the data to include only participants who responded using a smartphone device and re-examine the correlations between our context variables and the rmssd (i.e., we replicated Table 7, but only using smartphone responses). The results are virtually unchanged and are included below for the sake of completeness.

Task	Stress	Negative Affect	Social Interactions
Symbols	**83 [18,148]**	**134 [60,209]**	**-353 [-547, -159]**
Number	**92 [29,154]**	**147 [76,218]**	-180. [-374,14]
Mental	**110 [20,201]**	**154 [50,260]**	**-342 [-619,-65]**

For Review Only
